# Drought at a coastal wetland affects refuelling and migration strategies of shorebirds

**DOI:** 10.1007/s00442-021-05047-x

**Published:** 2021-10-16

**Authors:** Alexandra M. Anderson, Christian Friis, Cheri L. Gratto-Trevor, Christopher M. Harris, Oliver P. Love, R. I. Guy Morrison, Sean W. J. Prosser, Erica Nol, Paul A. Smith

**Affiliations:** 1 Environmental and Life Sciences Graduate Program, Trent University, Peterborough, Canada; 2 Canadian Wildlife Service, Environment and Climate Change Canada, Toronto, Canada; 3 Prairie and Northern Wildlife Research Centre, Environment and Climate Change Canada, Saskatoon, Canada; 4 Department of Integrative Biology, University of Windsor, Windsor, Canada; 5 National Wildlife Research Centre, Environment and Climate Change Canada, Ottawa, Canada; 6 Center for Biodiversity Genomics, University of Guelph, Guelph, Canada; 7 Department of Biology, Trent University, Peterborough, Canada

**Keywords:** Automated radio telemetry, Benthic invertebrates, DNA metabarcoding, Plasma metabolites, Standardised Precipitation-Evapotranspiration Index—SPEI

## Abstract

Droughts can affect invertebrate communities in wetlands, which can have bottom-up effects on the condition and survival of top predators. Shorebirds, key predators at coastal wetlands, have experienced widespread population declines and could be negatively affected by droughts. We explored, in detail, the effects of drought on multiple aspects of shorebird stopover and migration ecology by contrasting a year with average wet/dry conditions (2016) with a year with moderate drought (2017) at a major subarctic stopover site on southbound migration. We also examined the effects of drought on shorebird body mass during stopover across 14 years (historical: 1974–1982 and present-day: 2014–2018). For the detailed comparison of two years, in the year with moderate drought we documented lower invertebrate abundance at some sites, higher prey family richness in shorebird faecal samples, lower shorebird refuelling rates, shorter stopover durations for juveniles, and, for most species, a higher probability of making a subsequent stopover in North America after departing the subarctic, compared to the year with average wet/dry conditions. In the 14-year dataset, shorebird body mass tended to be lower in drier years. We show that even short-term, moderate drought conditions can negatively affect shorebird refuelling performance at coastal wetlands, which may carry-over to affect subsequent stopover decisions. Given shorebird population declines and predicted changes in the severity and duration of droughts with climate change, researchers should prioritize a better understanding of how droughts affect shorebird refuelling performance and survival.

**Supplementary Information:**

The online version contains supplementary material available at 10.1007/s00442-021-05047-x.

## Introduction

Droughts can substantially affect ecosystem structure and function. Reduced ground and surface water volume can change sedimentation and nutrient deposition (Baldwin and Mitchell 2000; Mishra and Singh 2010), reduce primary productivity (Zhao and Running 2010; Huang et al. 2016), and alter biological community composition to favour drought tolerant species (Chase 2007; Neto et al. 2010). Despite predictions of widespread and variable changes in the frequency and severity of droughts with climate change (Hoegh-Guldberg et al. 2018; Greve et al. 2018), little is known about responses to drought for many ecosystems. For higher trophic level organisms specifically, responses to drought are unknown for most ecosystems.

Coastal ecosystems are facing increasing pressures from climate change and development (Hoegh-Guldberg et al. 2018), and changes to drought conditions could put further strain on these ecosystems. The effects of drought on biota in estuarine systems are not well understood compared to other aquatic systems (Lake 2011). Most studies of drought at marine coastal areas have examined effects at lower trophic levels such as vegetation (McKee et al. 2004; Alber et al. 2008), zooplankton (Marques et al. 2007; Primo et al. 2009), meiofauna (Pillay and Perissinotto 2009), and benthic macrofauna (Pillay and Perissinotto 2008; Dittmann et al. 2015). These studies reported lower species richness and a dominance of saline tolerant taxa during and after drought (Pillay and Perissinotto 2009; Primo et al. 2009; Dittmann et al. 2015).

Little is known about how drought affects organisms at higher trophic levels in marine coastal ecosystems. Previous studies have largely focused on fish (Livingston 1997; Livingston et al. 1997; Wetz et al. 2011), whereas birds tend to be overlooked as predators in aquatic ecosystems (Steinmetz et al. 2003). Studies showing that droughts affect prey availability and, thus, avian predators at coastal ecosystems are primarily limited to large waterbirds during the breeding period (Bildstein et al. 1990; Gaines et al. 2000).

Shorebirds (Charadriidae and Scolopacidae), small- to medium-sized wading birds, are the primary vertebrate predators at many intertidal ecosystems (Mathot et al. 2018) and may also be affected by changes to prey availability during droughts. Invertebrate prey availability and abundance are critical for shorebird refuelling at stopover sites. Better refuelling conditions allow shorebirds to accumulate larger fuel loads at stopover sites, and larger fuel loads at departure have been associated with higher reproductive success and survival (Baker et al. 2004; Duijns et al. 2017; Rakhimberdiev et al. 2018). Given predicted increases in drought in some regions of the globe with climate change (Hoegh-Guldberg et al. 2018) and widespread population declines of shorebirds (Rosenberg et al. 2019; Smith et al. 2020), it is important to understand links between drought and shorebirds’ refuelling and migration behaviour at coastal stopover sites.

We conducted two studies at a major coastal stopover site in the subarctic during southbound migration. First, we conducted a detailed comparison of shorebird refuelling and migration behaviour in two years: a year with moderate, short-term drought and a year with average dry/wet conditions for the stopover site. In the two years, we explored how drought affected invertebrate prey abundance, shorebird diet diversity and composition, refuelling rates, stopover duration, and stopover decisions after departing the subarctic. Second, we used 14 years of data to examine the effects of drought on shorebird mass during stopover.

We hypothesized that drought would have bottom-up effects on shorebirds during stopover because of reduced invertebrate abundance. We predicted that year-to-year changes in invertebrate prey availability would result in diet changes and lower refuelling rates and mass for shorebirds, which, in turn, would affect shorebird stopover duration and subsequent stopover probability. We discuss how droughts at northern latitudes may influence patterns in shorebird abundance at more southerly stopover areas, which has implications for shorebird population monitoring.

## Materials and methods

### Study site

**Fig. 1:**
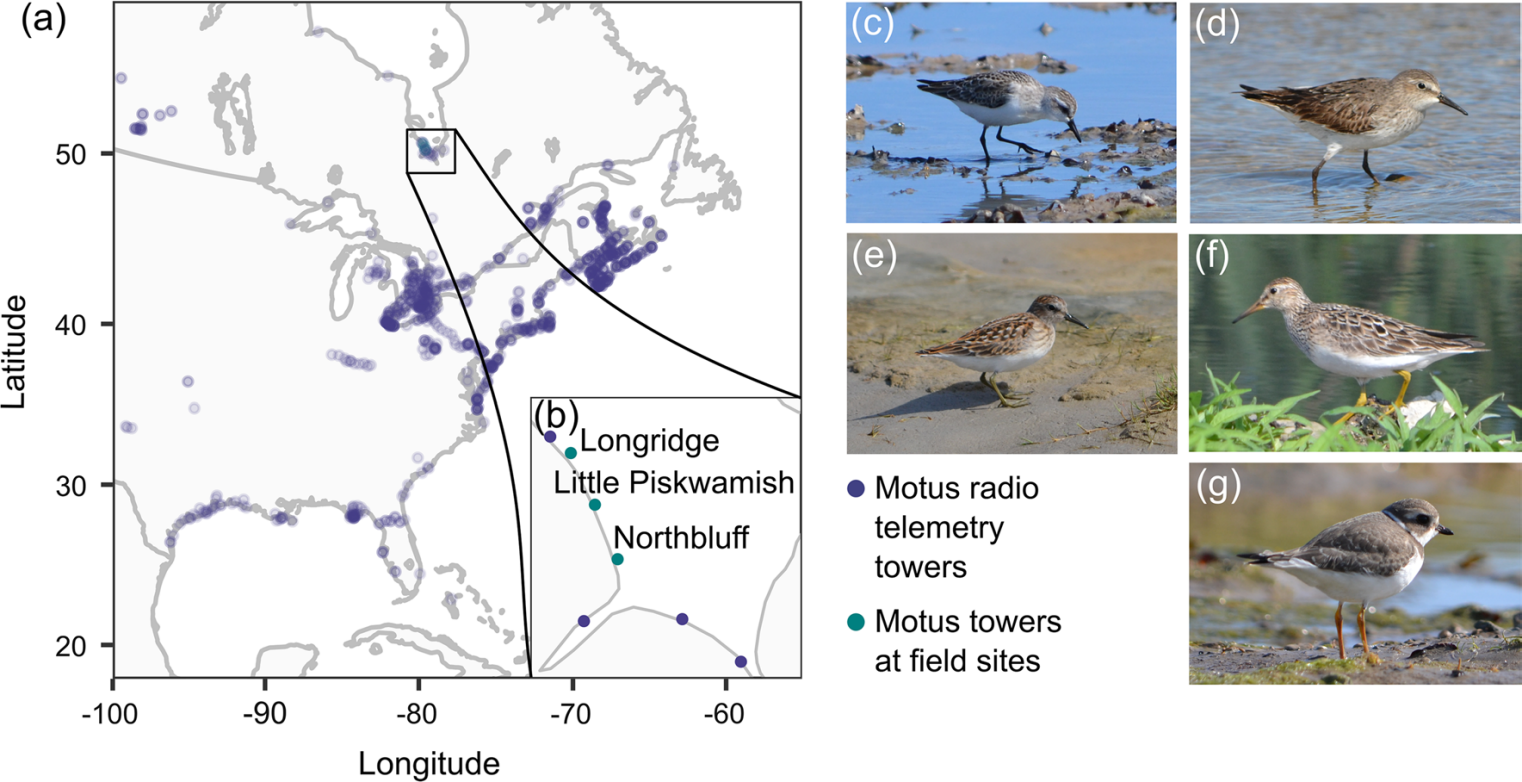
**a** Locations of Motus Wildlife Tracking System automated radio towers in 2016 and/or 2017. Darker points indicate multiple towers situated close together. **b** Radio towers along James Bay, Ontario, Canada. Labelled points designate field sites. Study species: **c** semipalmated sandpiper, **d** white-rumped sandpiper, **e** least sandpiper, **f** pectoral sandpiper, **g** semipalmated plover

We studied shorebirds along the southwestern coast of James Bay, Ontario, Canada (Fig. 1) during two time periods: 1974–1982 (see also Gratto 1983; Harrington and Morrison 1979; Morrison 1984) and 2014–2018, hereafter referred to as “historical” and “present-day”, respectively. This northern stopover site supports hundreds of thousands of shorebirds during southbound migration and is one of the first stopover sites used by shorebirds that breed in the eastern and central Canadian Arctic (Morrison 1984; McKellar et al. 2020). The study area is within the Hudson Bay Lowlands and consists of wide intertidal flats and coastal marshes bordered by the seasonally ice-covered waters of James Bay to the east and the boreal forest to the west.

### Drought index

We used the Standardized Precipitation-Evapotranspiration Index (SPEI; Beguería et al. 2014) as a measure of dry/wet conditions in the Hudson Bay Lowlands. The SPEI uses monthly mean temperature and precipitation data to estimate evapotranspiration and measure water balance (deficit and surplus) for a given location. The SPEI is measured in standard deviations (with most values between − 3 and 3), where negative values indicate periods of dryness and positive values indicate wet periods, both with reference to average conditions (SPEI = 0). For each year of the study, we sampled SPEI values from the Hudson Bay Lowlands (corresponding to 50.25° to 58.75° latitude and − 96.25° to − 77.25° longitude) using the SPEI Global Drought Monitor, which offers a spatial resolution of 1.0° lat/lon and a temporal resolution of 1 to 48 months (http://spei.csic.es/map/maps.html#months=1#month=3#year=2019). We averaged SPEI values spatially across the region and examined wet/dry conditions accumulating over a 3-month period (Jun–Aug) prior to and including the peak of southbound shorebird migration in August. We chose a 3-month time-period because it coincides with a surge in primary productivity (Glooschenko and Harper 1982; Cargill and Jefferies 1984) and the emergence and build-up of invertebrate prey biomass in the region after snow melt (Park 2017). Though adults tend to move through James Bay several weeks earlier than juveniles (Morrison 1984), we were interested in the broad, overall effects of drought accumulation throughout the subarctic growing season on shorebird refuelling. Therefore, we did not consider the effects of short-term, weekly changes in water balance that could occur (e.g., from a rainstorm) in James Bay between the times that adults and juveniles arrive.

### Shorebird banding

We captured shorebirds with mist nets (Gratto-Trevor 2004) at 11 banding locations situated at three field sites along the coast of James Bay (Fig. 1b) from mid-July through early September each year. Shorebirds were often captured during incoming tides (day and night) or at low tide in areas where nets were less visible (algal wracks, pools, or creeks). We weighed (historical: spring scales ± 0.5 g; present-day: electronic balance ± 0.1 g) and aged shorebirds as juveniles (< 1 year old, hatched several months prior) or adults (> 1 year of age, hatched in previous years) based on the shape and color of median wing coverts (Prater et al. 1977; Gratto-Trevor 2004). We examined the effects of drought on the 5 species that we captured most frequently: semipalmated sandpiper *Calidris pusilla* (Fig. 1c), white-rumped sandpiper *C. fuscicollis* (Fig. 1d), least sandpiper *C. minutilla* (Fig. 1e), pectoral sandpiper *C. melanotos* (Fig. 1f), and semipalmated plover *Charadrius semipalmatus* (Fig. 1g)*.*

### Detailed comparison of a year with average wet/dry conditions and a year with drought

We compared shorebird stopover and migration strategies during a “natural experiment” in drought conditions: a year with typical moisture conditions (2016) and a year with moderate drought (2017). In 2017, the Hudson Bay Lowlands was considered “abnormally dry” to experiencing a “moderate drought” (a drought that occurs every 3–5 or 5–10 years, respectively; Canadian Drought Monitor, Agriculture and Agri-Food Canada, 2017). Drought conditions persisted throughout Jun–Aug, which resulted in a 3-month SPEI value of − 0.93, a near-complete drying of supratidal marshes, and little to no freshwater flow into intertidal marshes. In contrast, wet/dry conditions were typical for the region during the previous year (2016: 3-month SPEI = 0.0007) with standing water in supratidal marshes and water flowing from small freshwater channels into the bay.

### Benthic invertebrate abundance

In 2016 and 2017, we sampled benthic macroinvertebrates at the three field sites during low tide (± 3 h) in two predominant habitat types used by shorebirds, intertidal marsh and intertidal flats, and in three habitat types available to shorebirds that covered less area: wrack, rock/pebble, and cyanobacteria mats (Online Resource, Table 1; Online Resource, Fig. 1). Within each habitat type, we opportunistically sampled invertebrates at locations where shorebirds were feeding (“foraging sites”) and at nearby (> 150 m) locations where there was no evidence of foraging (e.g., no shorebirds, bill marks, or footprints; “non-foraging sites”; Online Resource, Fig. 1). We sampled at non-foraging sites to better understand prey availability in the broader stopover habitat that shorebirds could use for foraging. Non-foraging sites appeared suitable for foraging but may have differed in some aspects of microhabitat, such as the amount of water saturation or sediment grain size.

At each foraging and non-foraging site, we collected three 5 cm diameter × 10 cm deep (196 cm^3^) benthic core samples and sieved each through 0.5 mm mesh. To sample wrack invertebrates, we filled the benthic coring device with algae and attempted to avoid compressing the algae beyond its natural density. We counted invertebrates from each core and identified them to family where possible. All prey taxa found in core samples (Online Resource, Fig. 2) were detected in shorebird faecal samples, except polychaetes.

In the two predominant habitat types for which we had suitable sample sizes (*n* > 30 per year), intertidal marsh and intertidal flats, we compared macroinvertebrate abundance between the two years using a generalized linear mixed effects model with a negative binomial distribution, which provided a better fit than a Poisson distribution (fitdistrplus package in R; Delignette-Muller and Dutang 2015). We used total invertebrate abundance per core as the response variable because most invertebrates were small enough for consumption by these species (Online Resource, Table 2), and DNA metabarcoding showed that shorebirds consumed a wide variety of invertebrates (see “Diet diversity and composition”). We considered the predictors year, habitat type (intertidal marsh or flats), day of year, site type (foraging or non-foraging), and interactions between year and site type, and year and habitat. We included random factors of site ID and paired site ID to control for replicate cores at each site and the proximity of foraging and non-foraging sites, respectively. We also included biologist ID as a random factor to account for different detection probability of invertebrate prey items by biologists during processing of core samples (*n* = 6). We included core volume (ml) as an offset to control for differences in sample volume when a full core could not be taken (e.g., because of the presence of rock or thick clay).

We conducted all analyses using program R version 3.6.2 (R Core Team 2019) and used the ‘drop1’ function with a Wald chi-square test (backwards stepwise approach) to remove variables that were not significant (*α* = 0.05 unless otherwise specified). We used the emmeans package (Lenth 2019) to estimate marginal effects (means or slopes while controlling for other model variables) and created figures with ggplot2 (Wickham 2016).

### Diet diversity and composition

We compared shorebird diet composition and diversity in 2016 and 2017 using DNA metabarcoding. During banding, we collected faecal samples from a subset of shorebirds (Online Resource, Table 3) by placing individuals in separate holding bins lined with clean wax paper. We stored faecal samples in 90% ethanol and at − 20 °C prior to homogenization and extraction (Moran et al. 2019). We amplified DNA using five primer pairs (Online Resource, Table 4), which targeted short regions of the mitochondrial gene cytochrome c oxidase subunit 1 (COI) of possible shorebird prey items: arthropods, amphipods, molluscs, annelids, and microalgae (e.g., diatoms). Diptera larvae, clams, and snails previously have been identified as prey items for shorebirds at James Bay (Morrison 1984), and amphipods, polychaetes, and biofilm (which contains large amounts of microalgae) are important prey items at other stopover sites (Hicklin and Smith 1984; Kuwae et al. 2012; Martínez-Curci et al. 2015).

We purified DNA following Moran et al. (2019) and sequenced it with an Ion Torrent S5 high-throughput sequencer (Thermo Fisher Scientific, Waltham, MA, USA) using a 530 chip according to the manufacturer’s instructions. We processed sequence reads through a bioinformatics pipeline (Prosser and Hebert 2017), which removed low quality reads (< 20 QV and < 100 bp) and excised primer and adapter sequences. We compared trimmed reads to the Barcode of Life Data System v4 (BOLD, www.boldsystems.org) reference library and assigned reads to operational taxonomic units (OTUs) using the BLAST algorithm. We only retained OTUs with at least ten reads. No prey items were detected in a subset of samples (19% of samples, *n* = 88), so they were removed from statistical analyses.

For each shorebird species, we compared prey family richness in faecal samples across the two years by calculating interpolation and extrapolation rarefaction curves of Hill numbers, which account for unequal sampling effort and incidence (presence/absence) data (Chao et al. 2014). For each species, we extrapolated data to two times the sample size of the year with the lowest sample size (Chao et al. 2014), and we used the iNEXT package (Hsieh et al. 2016) in R to calculate 95% confidence intervals by bootstrapping with 500 replications. We considered overlapping confidence intervals non-significant. We were not able to obtain enough samples to compare diets by age group (i.e., fewer than 5 samples per age group for multiple species in at least one year).

To compare broad differences in diet composition between years, we grouped prey items into ten major taxonomic groups (listed in Fig. 3) and compared frequency of occurrence of prey groups across years. We chose these taxonomic groupings because they were used in a previous study of shorebird diets using DNA metabarcoding (Gerwing et al. 2016) and represent large differences in possible prey items consumed (for example, bivalves vs. fly larvae instead of different families of fly larvae).

### Refuelling

We measured concentrations of three metabolites in shorebird blood plasma, triglyceride (TRIG), β-hydroxybutyrate (BUTY), and non-esterified fatty acid (NEFA), to compare shorebird refuelling rates between the two years. TRIG and BUTY concentrations tend to increase and decrease, respectively, with mass gain in birds (Williams et al. 1999; Cerasale and Guglielmo 2006), and NEFA levels may increase during exercise such as flight (McWilliams et al. 2004).

We drew blood from the brachial vein (75–150 μL) of a subset of shorebirds (Online Resource, Table 3) following Gratto-Trevor (2004) at Longridge and Northbluff field sites and recorded the time elapsed between capture and blood sampling (hereafter “bleed time”; median = 36 min) to account for changes in metabolites during the capture period (Guglielmo et al. 2002). We separated plasma from red blood cells by centrifugation (5000 RPM for 5 min) and froze samples in cryoshippers (− 150 °C) followed by freezers (− 80 °C). We measured metabolite concentrations (inter- and intra-assay coefficients of variation, Online Resource, Table 5) with commercially available kits (TRIG: #TR0100-1KT, Sigma-Aldrich, USA; BUTY: #K-HDBA, Megazyme, Ireland; NEFA: ELISA, FUJIFILM Wako Diagnostics, USA) optimized for use in shorebird and seabird species (Harris and Love, unpublished data).

We compared metabolite concentrations in the two years with MANCOVA models containing TRIG and BUTY (mmol L^−1^; log_e_ transformed) and NEFA (mEq L^−1^; log_e_ transformed) as response variables. We ran separate models for adults and juveniles because not all age classes were sampled for each species. Models contained fixed predictors of species, year, and an interaction between the two variables. We included body mass, time of day, day of year, bleed time, field site (Longridge or Northbluff), and tidal stage as covariates. We centred day of year and mass for each species and age class. To account for rhythmic and non-linear patterns in temporal variables, time of day and tidal stage were included as both sine and cosine terms (Bulla et al. 2017). We retained both sine or cosine terms in the model if either was significant (Hannon et al. 2018).We followed MANCOVA models with univariate tests for each metabolite to determine if annual differences in refuelling patterns were driven by one or more metabolites.

### Length of stay and subsequent stopover probability

We compared minimum stopover duration (length of stay after capture) of shorebirds in James Bay and subsequent stopover probability in North America using automated VHF radio telemetry. We attached digitally coded VHF avian nanotags (models ANTC-M4-2S, NTQB-3-2, and NTQB-4-2 with burst rates between 4.7 and 14.9 s and life spans of 90–170 days; Lotek Wireless, Newmarket, Ontario, Canada) to the lower back of a subset of shorebirds (Online Resource, Table 3) with cyanoacrylate glue (Loctite® Super Glue Control™ UltraGel™). Tags weighed 0.67 g or 1.3 g and did not exceed 5% of an individuals’ body mass. Tags were monitored by receiving stations at radio towers along James Bay (Fig. 1b), an antenna mounted to the base of a helicopter during the peak of the migratory period (once per year in early Aug), and through the Motus Wildlife Tracking System (Fig. 1a; Taylor et al. 2017).

We filtered telemetry data to remove probable false detections following Anderson et al. (2019). In brief, we removed detections with less than three consecutive bursts at intervals of a tag’s burst rate and detections on towers prone to noise. For individuals that we confirmed had departed James Bay (Anderson et al. 2019), we calculated minimum stopover duration in James Bay (time elapsed between capture and the last detection at James Bay). Because some shorebirds make non-stop flights from James Bay to South America (Brown et al. 2017), we classified individuals as to whether they stopped in North America or not, based on flight speeds and time spent in the vicinity of towers south of James Bay (Anderson et al. 2019). Individuals were considered to have made at least one stop in North America after departing James Bay if flight speeds were slow (< 9 m/s) between two consecutive towers (suggesting a stop occurred between the towers) or if detections at a tower or group of towers occurred for a longer period than the time it would take for a shorebird to fly past the detection area at a slow speed (9 m/s).

We compared length of stay at James Bay (in days as a continuous variable, log_e_ transformed) with linear models that contained species, year, capture day of year, and a species by year interaction as predictors. We assessed subsequent stopover probability with generalized linear models with a binomial response variable (stopped or did not stop) and species, year, and a species by year interaction. No semipalmated sandpiper adults made a subsequent stopover in North America in 2016; therefore, for this group, we considered the difference between 2016 and 2017 to be statistically significant if the coefficient for 2017 did not overlap zero. For both length of stay and stopover probability, we ran separate models for juveniles and adults because not all species were captured for each age class and age groups can have different migratory strategies (Newton 2011; Anderson et al. 2019). We included interaction terms in both models to determine if all species had the same pattern in stopover length and probability in the year with drought compared to the year with average conditions. We considered predictors of length of stay and stopover probability to be statistically significant at *α* = 0.10 because of low sample sizes of nanotagged birds (Online Resource, Table 3).

### Drought and shorebird body mass

We used linear models to determine if drought had consequences on shorebird body mass across multiple years. We ran separate models for each species because we could not reliably capture both age classes for all species, and sample sizes for some species were too low (*n* < 15) to analyze for some years (Online Resource, Table 6). We ran separate models by sex for pectoral sandpipers because, in contrast to other species considered in our study, pectoral sandpipers are more sexually dimorphic and can be differentiated by wing length (male flattened wing length > 140 mm, females < = 140 mm; Farmer and Wiens 1999). We log transformed (log_e_) the response variable (mass) and included predictors of age (adult or juvenile, if both age classes were captured), year-specific SPEI, and an interaction between the two variables. For all models, we included day of year as a covariate mean-centred for each age class to control for seasonal patterns in mass for each age group throughout the stopover period.

## Results

### Benthic invertebrate abundance

In the two years of detailed comparison, we sampled invertebrates at 225 shorebird foraging sites (Drought, 2017: *n* = 105; Average wet/dry, 2016: *n* = 120) and 228 non-foraging sites (Drought, 2017: *n* = 112; Average wet/dry, 2016: *n* = 116) and identified 16 families and 5 other taxa of invertebrates (Online Resource, Fig. 2). Invertebrate abundance was lower at non-foraging sites across habitats in the drought year (2017: mean ± SE: 1.4 ± 0.4 no. core^−1^) than the year with average wet/dry conditions (2016: 2.7 ± 0.7 no. core^−1^; *z* = 2.9, *P* = 0.004), but there was no difference in invertebrate abundance at foraging sites between the years (Drought, 2017: 2.2 ± 0.6 no. core^−1^; Average wet/dry, 2016: 2.7 ± 0.7 no. core^−1^; *z* = 0.9, *P* = 0.36). Invertebrate abundance was higher later in the season (1.3 ± 0.3 no. core^−1^ on 18-Jul compared to 3.45 ± 1.0 no. core^−1^ on 07-Sep; *z* = 3.6, *P* < 0.001) and at intertidal flats compared to intertidal marsh sites (flats: 3.8 ± 1.0 no. core^−1^; marsh: 1.25 ± 0.3 no. core^−1^; *z* = − 5.9, *P* < 0.001). Overall, invertebrate abundance was highest at wrack sites followed by intertidal flats (Online Resource, Fig. 3).

### Diet diversity and composition

**Fig. 2:**
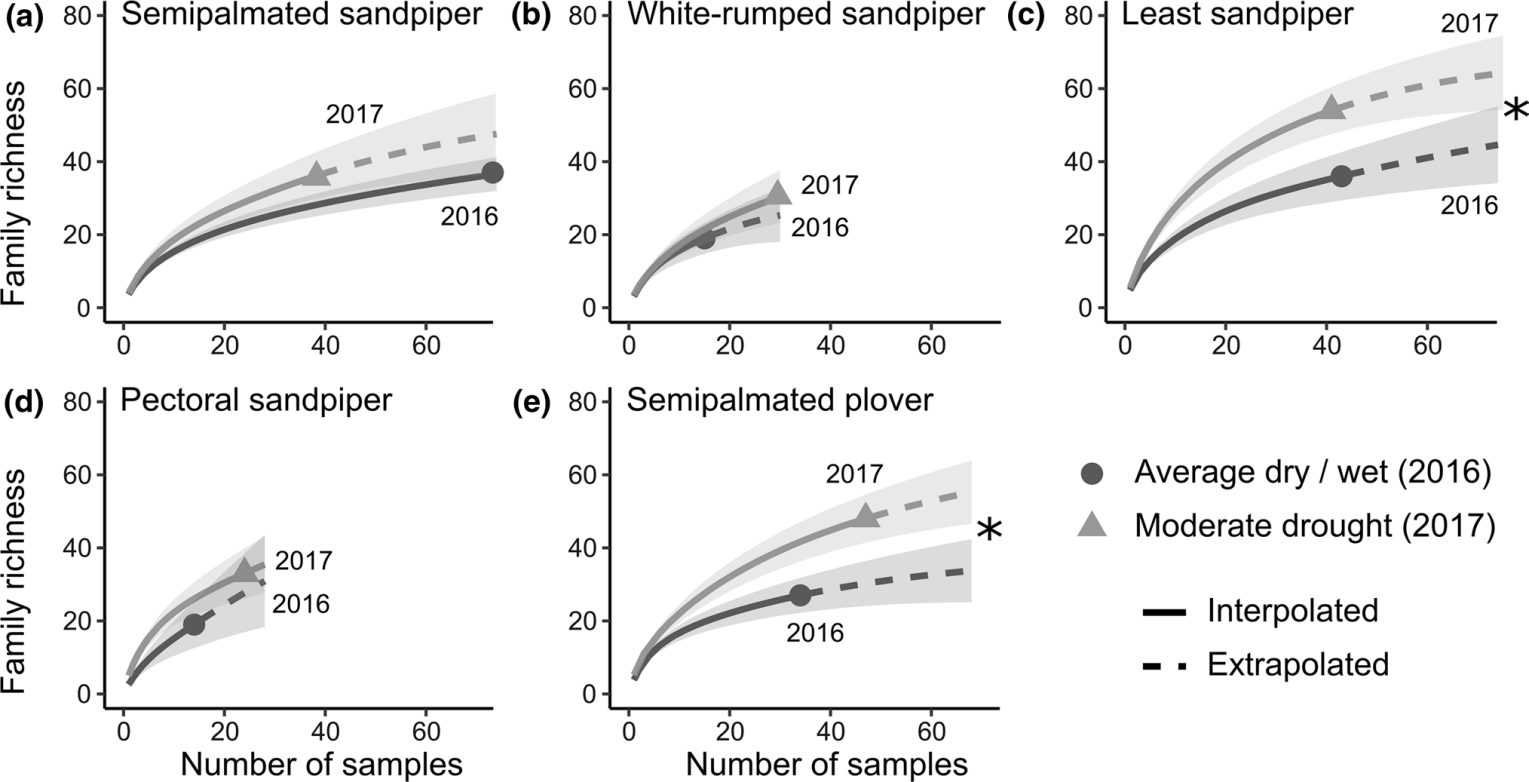
Family richness of prey items detected in shorebird faecal samples during a year with average wet/dry conditions (2016) and year with moderate drought (2017) at James Bay, Ontario, Canada. Shading represents 95% confidence intervals, and stars indicate statistical significance (*α* = 0.05)

**Fig. 3:**
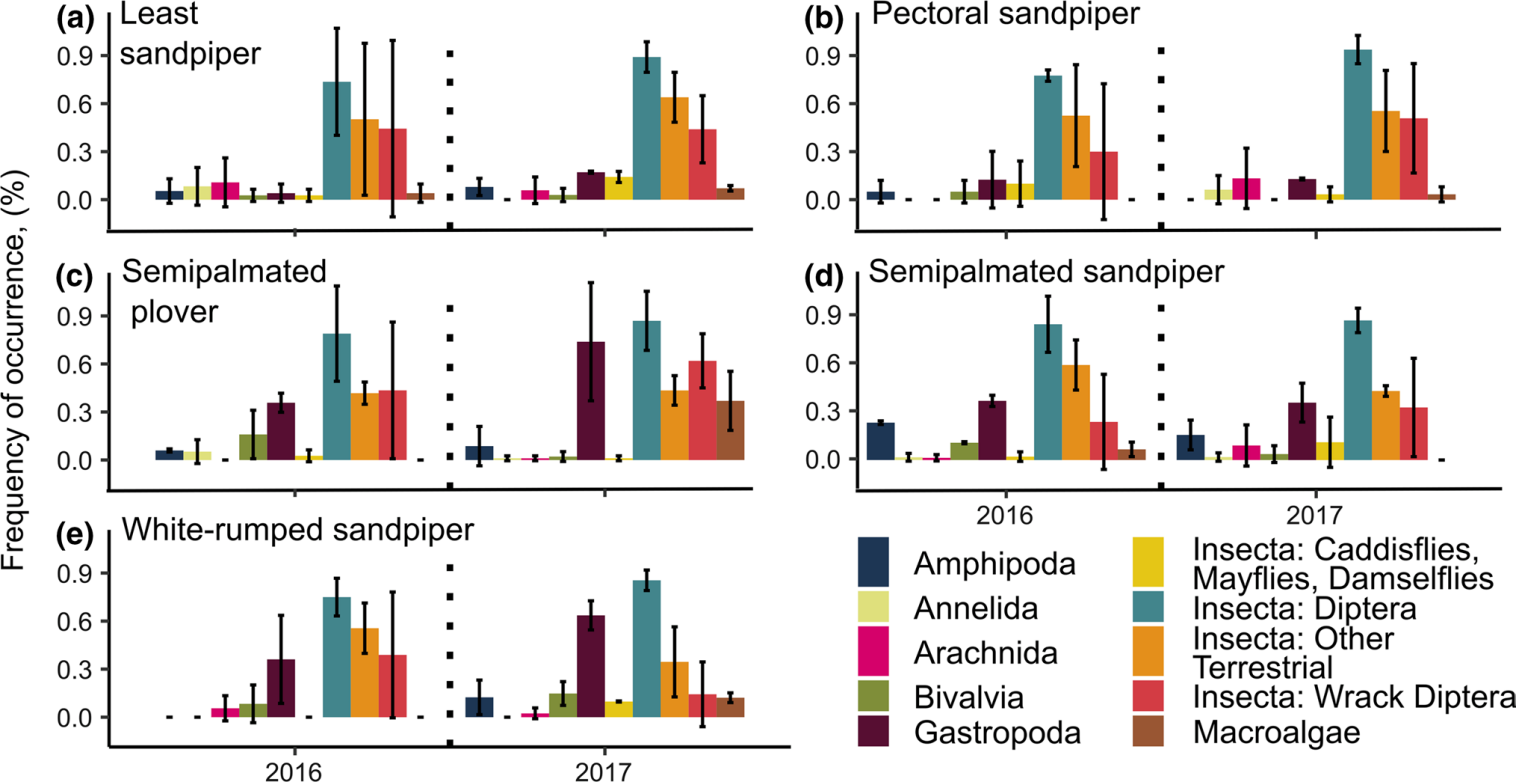
Frequency of occurrence of prey items from major prey groups in the faecal samples of shorebirds during a year with average dry/wet conditions (2016) and a year with moderate drought (2017) at James Bay, Ontario, Canada. No microalgae were detected. Values represent mean ± SD for samples collected at two habitat types (marsh and wrack)

Using DNA metabarcoding, we identified 101 families of prey items in shorebird faecal samples from 29 orders and 13 classes. For all species, prey family richness was higher in the year with drought compared to the year with average wet/dry conditions, but the relationship was only significant for least sandpipers and semipalmated plovers (Fig. 2). Prey family richness in faecal samples was similar among species (Fig. 2), and frequency of occurrence of prey groups was similar in the two years (Fig. 3).

### Refuelling

**Fig. 4:**
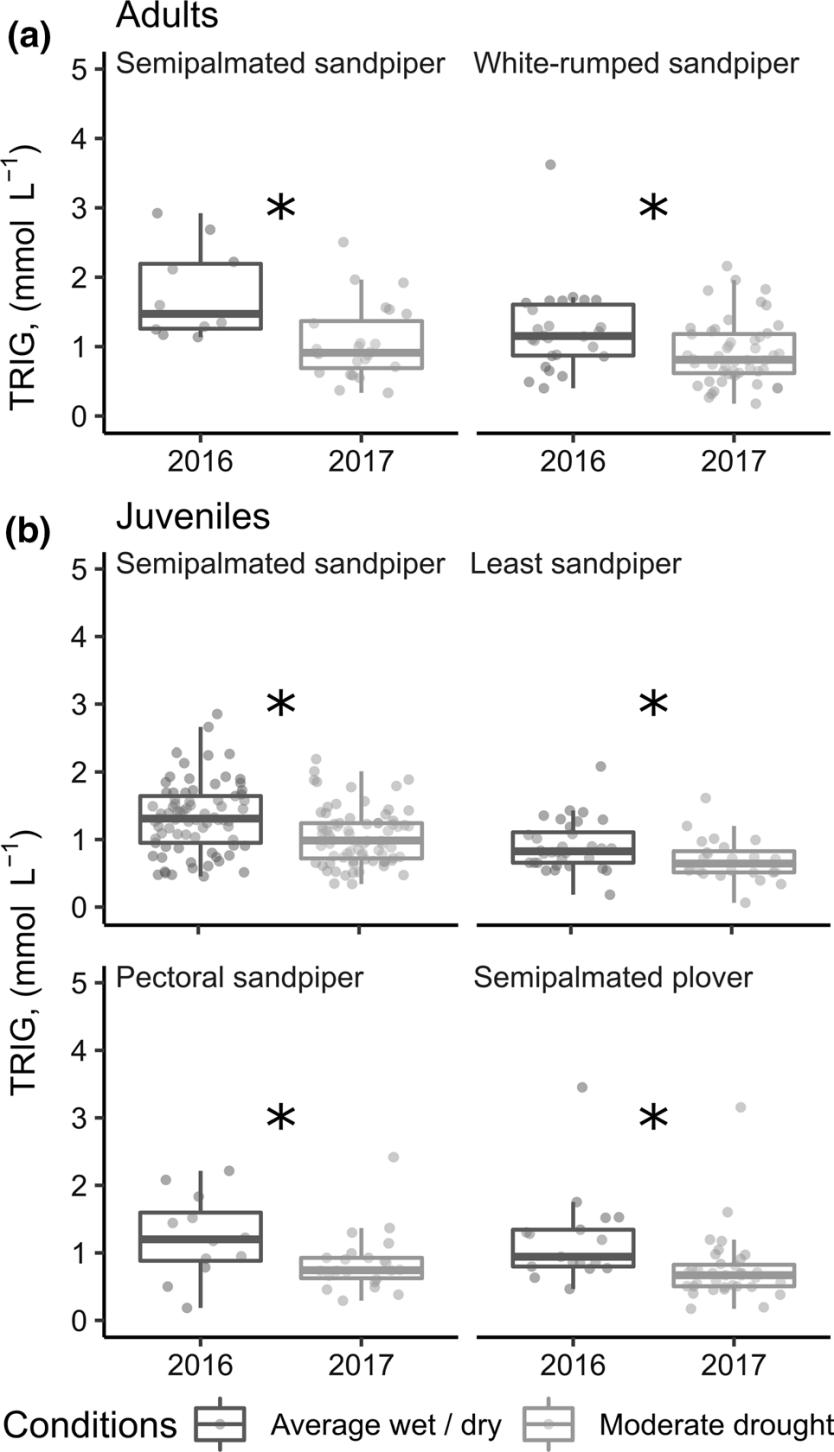
Plasma triglyceride concentrations for shorebirds at stopover along the southwestern coast of James Bay in a year with average wet/dry conditions (2016) and a year with moderate drought (2017). Boxplots and points represent raw data. Stars indicate a significant difference between years in univariate tests (*α* = 0.05)

For all species and age classes, we observed differences among plasma metabolite concentrations for the drought year versus the year with average wet/dry conditions (juveniles: Pillai’s trace = 0.09, *d.f.* = 1, *P* < 0.001; adults: Pillai’s trace = 0.35, *d.f.* = 1, *P* < 0.001). Higher concentrations of plasma TRIG were associated with lower concentrations of BUTY (Online Resource, Fig. 4 and 5). NEFA concentrations followed a similar pattern to BUTY concentrations (Online Resource, Fig. 4 and 5). Differences in plasma metabolite concentrations between the years were primarily driven by differences in TRIG concentrations (Online Resource, Table 7; Online Resource, Fig. 6), which were lower for all species and age classes during the year with drought (Fig. 4).

Body mass, bleed time, day of year, and time of day also explained plasma metabolite concentrations for juveniles and adults (Online Resource, Table 7). Refuelling rates were higher (higher TRIG, lower BUTY, lower NEFA) for juveniles captured later in the season compared to juveniles captured earlier in the season, whereas refuelling rates were lower for adults captured later (lower TRIG, higher BUTY; Online Resource, Table 7). TRIG concentrations were lowest in the morning and peaked in the evening for both age classes (Online Resource, Fig. 7).

### Length of stay and subsequent stopover probability

**Fig. 5:**
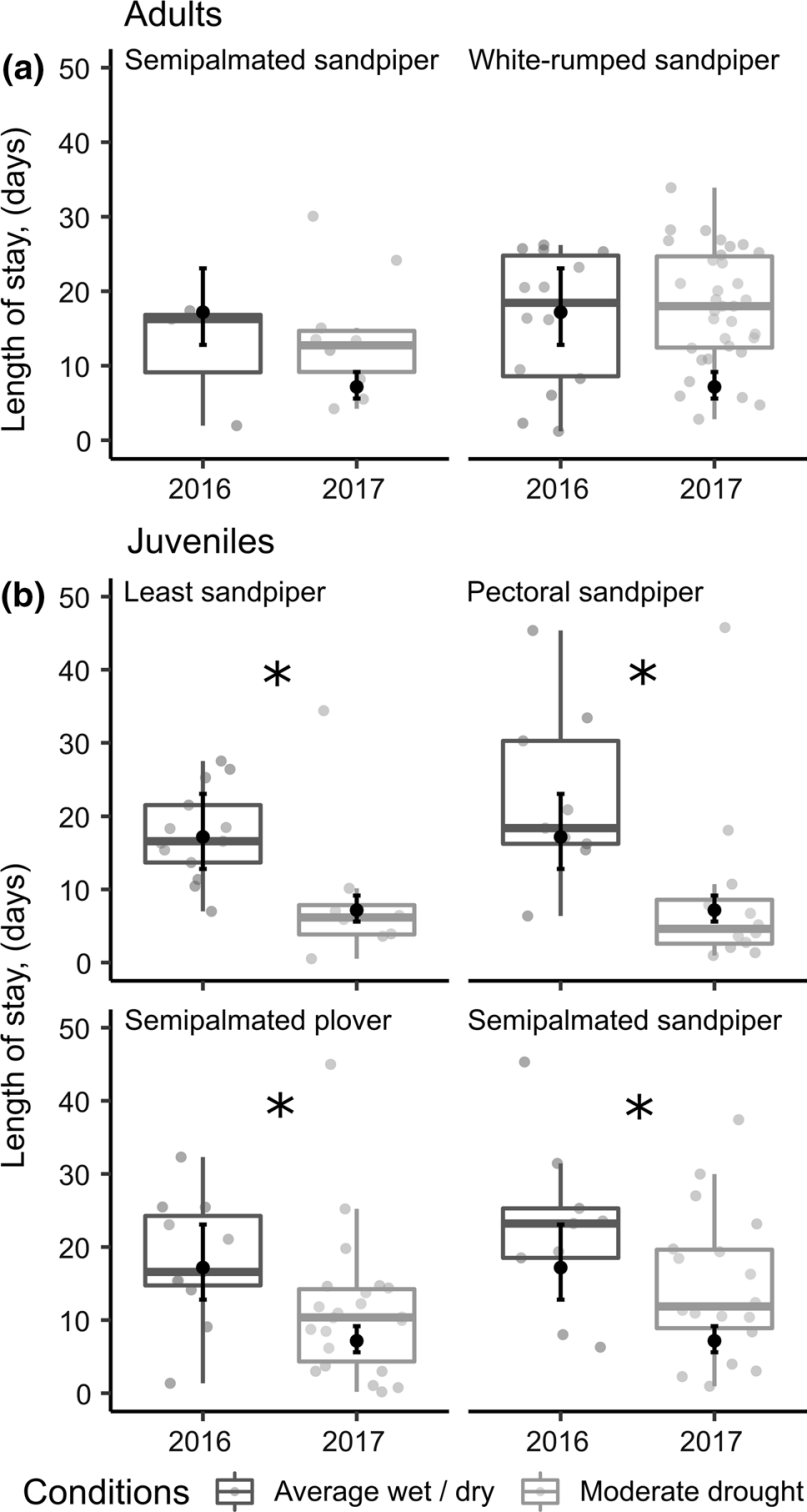
Minimum stopover duration of shorebirds at James Bay, Ontario, Canada in a year with average wet/dry conditions (2016) and a year with moderate drought (2017). Grey points and box plots represent raw data from VHF transmitters. Black points and error bars (95% CI) indicate back-transformed model predictions. Stars indicate statistical significance (*α* = 0.10)

Stopover lengths for adults at James Bay were the same during the two years (Fig. 5; semipalmated sandpiper: 13.4 ± 7.8 days and white-rumped sandpiper: 19.1 ± 11.5 days, global mean ± SD; *F*_1,51_ = 2.3, *P* = 0.13). Adult semipalmated sandpipers were more likely to make a subsequent stop in North America in the year with drought (Drought year, 2017: probability = 0.70 did not overlap with zero: 95% CI 0.38–0.90), but subsequent stopover probability did not differ between years for white-rumped sandpiper adults (Drought, 2017: 0.11 ± 0.05 probability ± SE; Average wet/dry, 2016: 0.23 ± 0.09; *z* = 1.20; *P* = 0.26).

Juveniles of all species stayed fewer days and less than half of the time in James Bay in the year with drought (2017: 7.2 ± 0.9 days; global mean ± SE) than the year with average conditions (2016: 17.2 ± 0.9 days; Fig. 5; *F*_1,100_ = 20.4, *P* < 0.001). Juveniles of all species were also more likely to make a subsequent stopover in North America in the year with drought (Drought, 2017: 0.52 ± 0.07; probability ± SE; Average wet/dry, 2016: 0.34 ± 0.08; χ^2^ = 3.0, *d.f.* = 1, *P* = 0.08). Stopover probability also differed by species (adults: χ^2^ = 7.3, *d.f.* = 1, *P* = 0.01; juveniles: χ^2^ = 11.9, *d.f.* = 3, *P* = 0.01).

### Drought and shorebird body mass

**Fig. 6:**
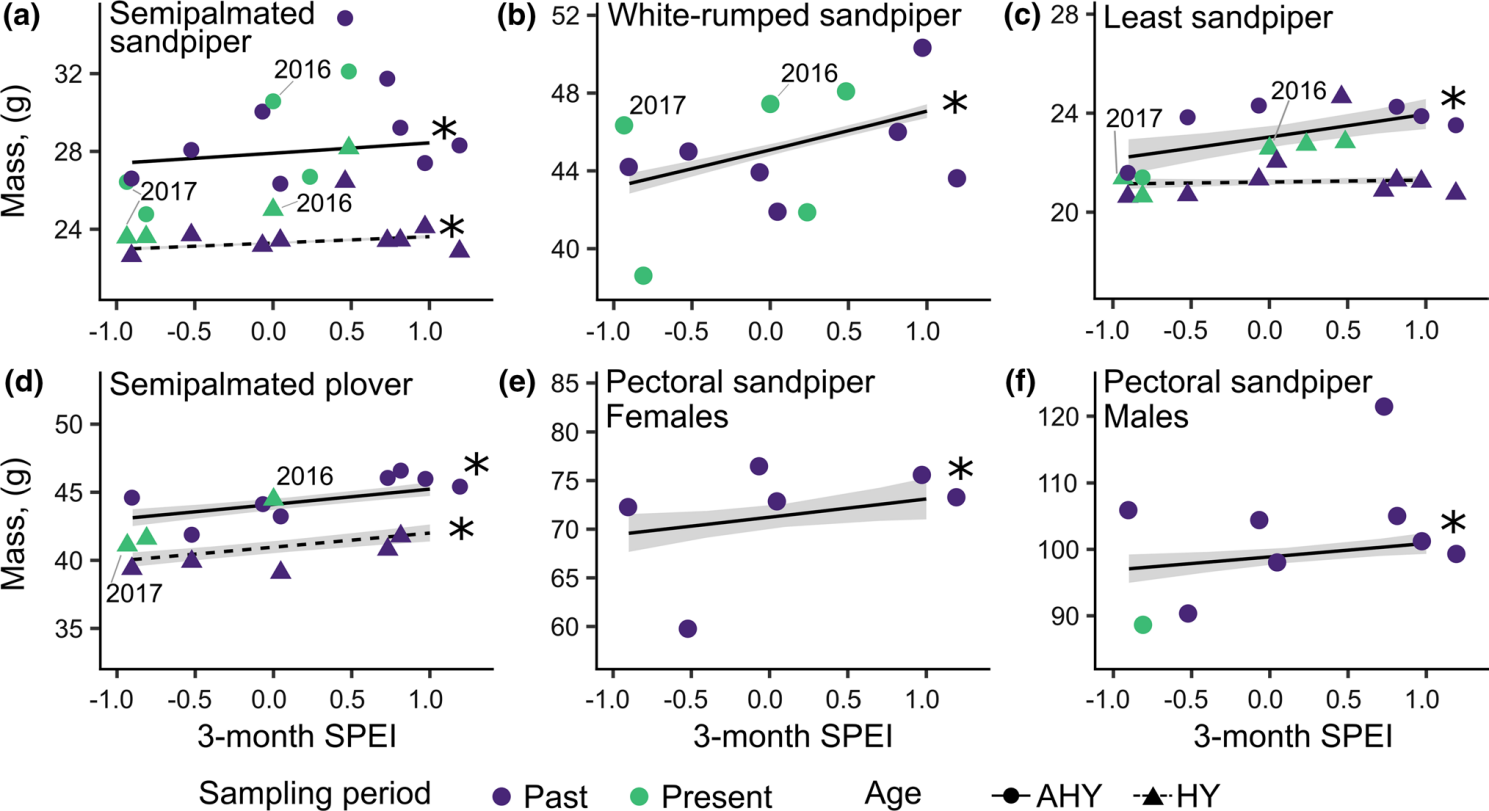
Model predicted effects of drought (95% CI back-transformed) on the mass of shorebirds during stopover along James Bay, Ontario, Canada. Solid lines represent adults, and dashed lines represent juveniles. SPEI − 1.0, 0, and 1.0 indicate moderate drought, average wet/dry conditions, and wet conditions, respectively, for the region. Points represent observed mean mass for a given species and age class in 1974–1982 (“past”, purple dots) and 2014–2018 (“present”, green dots). Years 2016 and 2017 (compared in-depth through further tests) are labelled for reference. Two points from the “past” sampling period for white-rumped sandpipers with mass greater than 52 g are not shown in the figure. Stars indicate a significant effect of SPEI on mass (*α* = 0.05)

We captured and measured the masses of > 50,000 shorebirds across the 14 years of the study (Online Resource, Table 8). Across these years, SPEI values in the Hudson Bay Lowlands ranged from − 0.93 (moderate drought) to 1.19 (wetter than average) (Fig. 6). Moderate droughts occurred in both the historical period (1981, SPEI = − 0.91) and the present-day (2017, SPEI = − 0.93). Across species, individuals had lower mass in drier years, after correcting for variation in capture date (Fig. 6). Body mass was ~ 1 to 8% lower across species and age groups for individuals in years with moderate drought (SPEI = − 0.9) compared to years that were moderately wet (SPEI = 1.0) (Table 1). Juveniles weighed less than adults across species (Fig. 6), but the slope of the relationship between SPEI and mass was the same for each age group across species (except juvenile least sandpipers, for which there was no relationship between mass and SPEI; Online Resource, Table 8).

**Table 1:** Model predicted estimates of shorebird mass during moderate drought (Standardized Precipitation Index, SPEI = − 0.9) and abnormally wet (SPEI = 1.0) periods at James Bay, Ontario, Canada

Species	Estimated mass (g)				~ % body mass difference	
	SPEI -0.9		SPEI 1.0			
	Juv	Ad	Juv	Ad	Juv	Ad
Least sandpiper	21.1	22.2	21.3	24.0	0.9	8.1
Semipalmated sandpiper	23.0	27.4	23.6	28.4	2.6	3.6
White-rumped sandpiper		43.4		47.1		8.5
Semipalmated plover	40.4	43.1	42.0	45.2	4.7	4.9
Pectoral sandpiper females		69.6		73.1		5.0
Pectoral sandpiper males		97.1		101.0		3.9

Mass at low and high SPEI levels was estimated from models during the mean capture day of year during stopover for each species and age class (Juv = juveniles; Ad = adult). % body mass difference is the percent change in mass for each age class between SPEI − 0.9 and SPEI 1.0

## Discussion

Moderate, short-term drought was associated with changes in refuelling and migration behaviour across a variety of Arctic breeding shorebirds at a key stopover site during southbound migration. In a detailed comparison of a year with moderate drought and a year with average wet/dry conditions, moderate drought was linked with lower invertebrate abundance at some sites, higher diet diversity, lower refuelling performance (i.e., plasma triglycerides), and shorter stopover duration for juveniles. Additionally, for most species and age classes, telemetry detections suggested that individuals were more likely to make a stopover in North America after departing the subarctic in the year with drought than take a non-stop flight to South America. In a 14-year dataset, shorebirds had lower body mass in drier years, which provides further support for the hypothesis that drought affects shorebird refuelling at coastal stopover sites. To our knowledge, our study is the first to examine the effects of drought on shorebird stopover ecology at a coastal stopover site and the first to show a possible carry-over effect of drought conditions on subsequent stopover probability of shorebirds.

Lower refuelling rates for shorebirds have previously been associated with low prey abundance at stopover sites (Acevedo Seaman et al. 2006). We observed lower invertebrate abundance at non-foraging sites during the drought year. This result paired with lower triglyceride concentrations in shorebird blood plasma and lower shorebird body mass in drier years provides support for the hypothesis that drought effects prey availability and shorebird refuelling at coastal stopover sites. Although we did not systematically sample invertebrates across the same sites in both years, our methods for selecting and sampling at non-foraging sites did not differ between the two years, which suggests that invertebrate abundance was lower in some areas in the year with drought. Lower invertebrate abundance at non-foraging sites may have resulted from increased salinity and prolonged sediment exposure during drought (Dittmann et al. 2015), which can lead to desiccation and/or osmotic pressure imbalance and, ultimately, mortality of invertebrates (Sutcliffe 1961; Kapoor 1978). Similarly, reduced freshwater outflow from marshes can limit the allochthonous nutrient inputs to the intertidal environment (Elliott and Whitfield 2011) and lead to nutrient stress for invertebrates (Lake 2008, 2011).

In contrast to non-foraging sites, we observed equal densities of invertebrates at shorebird foraging sites in the two years of the study. Despite locating areas with the same prey density in the two years, shorebird refuelling rates still were lower during the year with moderate drought than the year with average dry/wet conditions. Lower refuelling rates may have resulted from increased competition and lower prey intake rates at foraging sites (Hake and Ekman 1988). Shorebirds also may have had difficulty reaching benthic invertebrates, which may migrate deeper into the sediment to avoid desiccation during drought (Williams 1977; Frouz et al. 2003). Regardless of the mechanism, reduced refuelling performance at stopover sites has been linked to lower survival in shorebirds (Baker et al. 2004; Duijns et al. 2017; Rakhimberdiev et al. 2018). Therefore, reduced refuelling performance during droughts could reduce shorebird survival and contribute to population declines.

Because macroinvertebrate diversity can decrease during drought (Dittmann et al. 2015), we were surprised to see a pattern of higher prey family richness in diets of some shorebird species during the year with drought compared to the year with average wet/dry conditions. More diverse diets during the drought year could result from individuals consuming alternative prey items if preferred prey families were less available (Thompson and Colgan 1990; Svanbäck and Bolnick 2007). Although generalist diets may have evolved to exploit heterogeneous environments (Levins 1968; Sexton et al. 2017), higher prey family richness in shorebird diets in the drought year was paired with lower refuelling rates and body mass. Lower refuelling rates and body mass during drought could indicate that broader diets were not sufficient to overcome lower prey abundance, perhaps because less favourable prey items (e.g., lower nutritional value or more difficult to digest) were selected during the drier year.

Lower shorebird refuelling rates were paired with shorter stopover duration for juveniles, but not adults, in James Bay in the year with moderate drought. Juveniles may stop for a shorter period of time at sites with poor foraging conditions in hopes of finding better conditions elsewhere (e.g., “the expectation rule”, Alerstam 2011), whereas adults may stop for a constant amount of time or stay at a stopover site with poor conditions because they anticipate poor foraging conditions throughout the migratory range (e.g., “the global update rule”, Alerstam 2011). Alternatively, as more experienced foragers than juveniles, adults may have taken advantage of sudden increases in prey availability (e.g., invertebrates moving closer to the sediment surface during short periods of rain), allowing for suitable mass gain despite drought conditions. We have some evidence that adult shorebirds gained suitable weight (e.g., 33–35 g for semipalmated sandpipers, Gratto 1983) in drier years during the historical period (Online Resource, Fig. 8), but we do not have sufficient recapture data from the present-day to determine if this still occurs.

In addition to shorter stopovers, patterns in flight speeds and transmitter detections suggested that juveniles were more likely to make a subsequent stopover in North America after departing the subarctic in the year with moderate drought than the year with typical wet/dry conditions. A similar pattern of higher stopover probability in the drought year was observed for semipalmated sandpiper adults. Interestingly, we found a higher probability of stopover in the year with drought across a variety of shorebirds with different migratory strategies (Anderson et al. 2019), except for white-rumped sandpipers which are extreme long-distance migrants and may have less flexible migratory behaviours.

We cannot rule-out the possibility that subsequent stopovers in North America went undetected during the drought year; for example, more shorebirds may have stopped in the southern United States where Motus towers are sparse during the drought year than the year with average conditions. We think this is unlikely because resights of hundreds of marked shorebirds from James Bay on southbound migration are almost exclusively in Atlantic Canada and the northeastern United States (Morrison 1984) where Motus tower density is high. Future tracking efforts, such as expansion of the Motus Wildlife Tracking System into the southern United States, could help clarify the extent that shorebirds migrating from James Bay use these southern stopover sites in the United States.

Few studies have examined how drought conditions carry-over to affect subsequent stopover decisions in birds, and our study is the first to observe a higher stopover probability after departure from a site with drought conditions. Previous studies of songbirds at wintering areas have linked dry conditions to delays in migratory departure, shorter total migration distances, and delayed arrival at breeding areas (Studds and Marra 2011; Tøttrup et al. 2012; McKinnon et al. 2015). Our results suggest that drought conditions at key stopover sites may lead to similar carry-over effects on subsequent life history stages of the annual life cycle for shorebirds.

Our finding that refuelling conditions in the subarctic may carryover to influence whether shorebirds stop at additional sites in North America could help explain regional and annual patterns in shorebird abundance in Atlantic Canada and the northeastern United States. Counts of shorebirds at temperate stopover sites are often used to assess shorebird population trends (for example, through the Atlantic Canada Shorebird Survey or International Shorebird Survey, Rosenberg et al. 2019). Accounting for conditions in the subarctic (i.e., drought), and, therefore, the possibility of shorebirds stopping or by-passing survey areas, could help to refine estimates of population trends determined from these surveys.

Understanding the effects of drought on shorebird refuelling and migration behaviour is essential given changing global patterns in dry and wet conditions (Hoegh-Guldberg et al. 2018) and widespread declines of Arctic breeding shorebirds (Rosenberg et al. 2019; Smith et al. 2020). Though the Arctic is predicted to become more wet on average with climate change (Hoegh-Guldberg et al. 2018; Greve et al. 2018), summers (Jun–Aug) are predicted to become drier in the Canadian subarctic (Tam et al. 2019). Our study shows that even moderate drought conditions at a key coastal stopover site in the Canadian subarctic are associated with lower body mass, refuelling rates, and changes in migration for shorebirds. Increases in the frequency or severity of dry conditions in the region could worsen refuelling conditions for shorebirds, which is concerning because shorebirds that do not acquire high fuel loads at stopover sites may be less likely to survive (Baker et al. 2004; Duijns et al. 2017; Anderson et al. 2019). Given predictions of increased severity and duration of droughts at some regions with climate change, researchers should prioritize understanding how these dry periods affect shorebird refuelling performance and if they contribute to population declines of shorebirds.

## Supplementary Information

Below is the link to the electronic supplementary material.

Supplementary file1 (DOCX 2609 kb)

## Data Availability

SPEI data used in this study are publicly available at: http://spei.csic.es/map/maps.html#months=1#month=3#year=2019. Data from the Canadian Drought Monitor are publicly available from Agriculture and Agri-Food Canada at: https://open.canada.ca/data/en/dataset/292646cd-619f-4200-afb1-8b2c52f984a2. Automated radio telemetry data are stored at https://motus.org/. Invertebrate sampling, shorebird mass, DNA metabarcoding, plasma metabolite, length of stay, and stopover data are publicly accessible without restriction at: https://osf.io/vpm7f/?view_only=ea3f6477e584428d8bdca78b978a8653.
