# Supplementary material for: Drought at a coastal wetland affects refuelling and migration strategies of shorebirds
Source: Oecologia. 2021 Oct 16;197(3):661–74. doi: 10.1007/s00442-021-05047-x (PMC8585834; doi:10.1007/s00442-021-05047-x)
Supplement: Supplementary file 1 — Supplementary file1 (DOCX 2609 kb) [file 442_2021_5047_MOESM1_ESM.docx]

**Drought at a coastal wetland affects refuelling and migration strategies of shorebirds**

**Anderson, Friis, Gratto-Trevor, Harris, Love, Morrison, Prosser, Nol, and Smith**

**Electronic Supplementary Material**

| **Supplementary Table 1.** Descriptions of habitat types where of benthic core samples were collected at James Bay, Ontario, Canada in 2016 and 2017. | | |
| --- | --- | --- |
| Cyanobacteria mat | 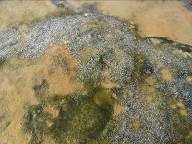 | Blue-green algae and bacteria that form thick, gelatinous mats in intertidal marshes |
| Intertidal flats | 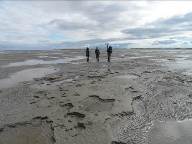 | Flats of sand and mud which extends approximately ~1600 m during low tide |
| Intertidal marsh | 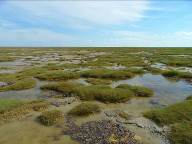 | A transitional habitat between supratidal marsh and intertidal flats that is subject to tidal inundation and characterized by a patchy network of vegetation, especially *Puccinellia phryganodes* |
| Rock/pebble | 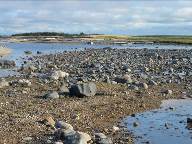 | Sediment consisting primarily of rocks or pebbles |
| Wrack | 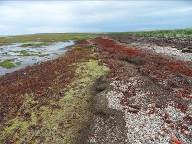 | Beach-cast materials, especially dulce red algae *Palmaria palmata*, that wash up on small ridges |

| **Supplementary Table 2.** Length of a subset of benthic macroinvertebrates sampled at Northbluff Point, James Bay, Ontario in 2017. *Macoma balthica* were measured at the widest part of the shell. Invertebrates were identified to species where possible. * indicates that a species or genus was identified by DNA metabarcoding through the International Barcode of Life Barcode 500K program <https://ibol.org/programs/barcode-500k/>. Almost all polychaetes found during the study were small *Spio* polychaetes similar to the individual measured here. | | | | | |
| --- | --- | --- | --- | --- | --- |
|  |  | length (mm) | | | |
| family | species | median | mean | sd | *n* |
| Ceratopogonidae |  | 5.0 | 5.3 | 0.5 | 4 |
| Chironomidae |  | 4.5 | 4.6 | 1.3 | 10 |
| Dolichopodidae |  | 4.5 | 4.5 | 1.1 | 6 |
| Ephydridae |  | 3.0 | 2.8 | 0.7 | 9 |
| Hydrobiidae | *Ecrobia truncata* | 1.5 | 1.7 | 1.3 | 62 |
| Naididae | *Paranais litoralis ** | 5.0 | 5.3 | 2.2 | 29 |
| Scathophagidae |  | 5.0 | 5.0 | NA | 1 |
| Spionidae | *Spio sp.* * | 5.0 | 5.0 | NA | 1 |
| Tellinidae | *Macoma balthica* | 2.0 | 3.9 | 3.3 | 628 |

| **Supplementary Table 3.** Sample sizes of shorebirds included in comparative analyses of a year with average dry/wet conditions (2016) and a year with moderate drought (2017). | | | | | | | | | | | |
| --- | --- | --- | --- | --- | --- | --- | --- | --- | --- | --- | --- |
|  |  | least sandpiper | | semipalmated sandpiper | | white-rumped sandpiper | | semipalmated plover | | pectoral sandpiper | |
|  |  | 2016 | 2017 | 2016 | 2017 | 2016 | 2017 | 2016 | 2017 | 2016 | 2017 |
| adult | plasma metabolites (MANCOVA) |  |  | 10 | 12 | 16 | 20 |  |  |  |  |
|  | nanotagged |  |  | 7 | 14 | 25 | 45 |  |  |  |  |
|  | nano (length of stay) |  |  | 3 | 10 | 14 | 36 |  |  |  |  |
|  | nano (future stopover probability) |  |  | 5 | 10 | 22 | 35 |  |  |  |  |
|  | faecal | 1 | 4 | 4 | 15 | 14 | 29 | 5 | 3 | 2 | 8 |
|  |  |  |  |  |  |  |  |  |  |  |  |
| juvenile | plasma metabolites (MANCOVA) | 20 | 12 | 55 | 57 |  |  | 13 | 24 | 11 | 21 |
|  | nanotagged | 21 | 19 | 23 | 32 |  |  | 14 | 29 | 12 | 19 |
|  | nano (length of stay) | 13 | 8 | 9 | 18 |  |  | 11 | 22 | 9 | 12 |
|  | nano (future stopover probability) | 16 | 10 | 14 | 24 |  |  | 10 | 19 | 7 | 8 |
|  | faecal | 42 | 36 | 96 | 23 | 1 | 2 | 29 | 44 | 11 | 16 |

| **Supplementary Table 4.** Primer pairs and associated thermocycling conditions used to amplify prey DNA in shorebird faecal samples. All primers were previously unpublished except for the arthropod primer which was published by Zeale et al. (2011). | | | | |
| --- | --- | --- | --- | --- |
| target taxa | forward primer sequence  (5’ 🡪 3’) | reverse primer sequence  (5’ 🡪 3’) | amplicon length (bp) | thermocycling |
| arthropods | AGATATTGGAACWTTATATTTTATTTTTGG | GGAGGATTTGGWAATTGATTAGTW | 157 | 53°C, 60 cycles, 30 sec extension |
| molluscs | GCTGGWRTWTCTTCWATTYTTG | AATTTTAAYACYTCTTTYTTTG | 161 | 45°C, 20 cycles then 51°C, 40 cycles, 30 sec extension |
| annelids | GTAGATYTNGCWATTTTYTC | AAYYTWAAYACWDCTTTTTTTG | 193 | 45°C, 20 cycles then 51°C, 40 cycles, 30 sec extension |
| amphipods | GTAGATYTNGCWATTTTYTC | AAYHTWAAYACHTCTTTTTTTG | 193 | 45°C, 20 cycles then 51°C, 40 cycles, 30 sec extension |
| microalgae | GATTTAGCWATTTTTAGTTTRCAT | ATGYTDTTAACWGATAGAAATT | 168 | 45°C, 20 cycles then 51°C, 40 cycles, 30 sec extension |

| **Supplementary Table 5.** Inter- and intra-assay coefficients of variation of triglyceride (TRIG), β-hydroxybutyrate (BUTY), non-esterified fatty acid (NEFA) concentration measurements from shorebird blood plasma. We ran samples in duplicate (TRIG, NEFA) or triplicate (BUTY) and retained the average values for analyses. | | | | |
| --- | --- | --- | --- | --- |
| metabolite | year | plates (*n*) | inter-assay CV % | intra-assay CV % |
| TRIG | 2016 | 5 | 15.42% | 4.91% |
|  | 2017 | 7 | 9.09% | 4.49% |
| BUTY | 2016 | 8 | 8.45% | 3.38% |
|  | 2017 | 10 | 7.81% | 3.86% |
| NEFA | 2016 | 4 | 6.69% | 2.91% |
|  | 2017 | 5 | 10.50% | 3.80% |

| **Supplementary Table 6.** Sample sizes of shorebirds included in analyses of drought effects on body mass. Species with *n* < 15 per age class (ad = adult, juv = juvenile or sex for pectoral sandpipers) in a year were excluded from analyses. | | | | | | | | | | | | |
| --- | --- | --- | --- | --- | --- | --- | --- | --- | --- | --- | --- | --- |
|  | least sandpiper | | semipalmated sandpiper | | white-rumped sandpiper | | semipalmated plover | | pectoral sandpiper | | | |
|  |  |  |  |  |  |  |  |  | females | | males | |
| year | ad | juv | ad | juv | ad | juv | ad | juv | ad | juv | ad | juv |
| 1974 |  | 18 | 28 | 198 | 17 |  |  |  |  |  |  |  |
| 1975 |  | 70 | 2087 | 508 | 589 |  | 37 | 15 |  |  | 18 |  |
| 1976 | 24 | 258 | 6019 | 2857 | 402 |  | 66 | 47 | 36 |  | 45 |  |
| 1977 | 52 | 575 | 5347 | 3322 | 532 |  | 100 | 113 |  |  | 37 |  |
| 1978 | 24 | 162 | 5021 | 451 | 480 |  | 84 |  | 64 |  | 142 |  |
| 1979 |  | 520 | 2265 | 1770 | 221 |  | 102 | 45 | 168 |  | 192 |  |
| 1980 | 37 | 280 | 1376 | 1206 | 157 |  | 48 |  | 59 |  | 77 |  |
| 1981 | 23 | 211 | 1355 | 3216 | 159 |  | 72 | 39 | 90 |  | 96 |  |
| 1982 | 25 | 303 | 1858 | 1031 | 147 |  | 49 |  | 30 |  | 134 |  |
| 2014 |  | 15 | 227 |  | 71 |  |  |  |  |  |  |  |
| 2015 |  | 91 | 103 | 148 | 72 |  |  |  |  |  |  |  |
| 2016 |  | 130 | 110 | 474 | 64 |  |  | 41 |  |  |  |  |
| 2017 |  | 76 | 46 | 435 | 64 |  |  | 64 |  |  |  |  |
| 2018 | 18 | 40 | 120 | 241 | 50 |  |  | 24 |  |  | 16 |  |

| **Supplementary Table 7.** Results from MANCOVA models and post-hoc univariate tests examining predictors of three plasma metabolite concentrations (TRIG, BUTY, and NEFA) in shorebird blood plasma at James Bay, Ontario, Canada in 2016 and 2017. Estimates are presented on the log scale, listed in reference to year 2016 and, for juveniles, in reference to least sandpipers. Species was removed from the final MANCOVA model for adults. Significant effects (*α* = 0.05) are bolded. | | | | | | | | | | | | | | |
| --- | --- | --- | --- | --- | --- | --- | --- | --- | --- | --- | --- | --- | --- | --- |
|  |  | | | TRIG | | |  | BUTY | | |  | NEFA | | |
| **adults** | df | Pillai’s Trace | multi- variate *p* | *β* | SE | uni- variate *p* |  | *β* | SE | uni- variate *p* |  | *β* | SE | uni- variate *p* |
| intercept |  |  |  | 0.56 | 0.13 |  |  | -1.17 | 0.23 |  |  | 0.58 | 0.10 |  |
| day of year | 1 | 0.28 | **< 0.001** | -0.01 | 0.01 | **< 0.001** |  | 0.01 | 0.01 | **0.03** |  | -0.02 | 0.005 | **< 0.001** |
| year 2017 | 1 | 0.35 | **< 0.001** | -0.43 | 0.10 | **< 0.001** |  | 0.03 | 0.17 | 0.97 |  | 0.04 | 0.07 | 0.63 |
| body mass | 1 | 0.34 | **< 0.001** | 0.04 | 0.01 | **< 0.001** |  | -0.03 | 0.01 | **0.03** |  | 0.002 | 0.01 | 0.98 |
| bleed time | 1 | 0.26 | **< 0.01** | -0.005 | 0.002 | **< 0.001** |  | 0.01 | 0.003 | **< 0.001** |  | 0.001 | 0.001 | 0.06 |
| sin (time of day) | 1 | 0.16 | **0.04** | -0.12 | 0.12 | 0.65 |  | 0.20 | 0.19 | 0.44 |  | 0.24 | 0.08 | **< 0.01** |
| cos (time of day) | 1 | 0.23 | **< 0.01** | 0.34 | 0.09 | **< 0.001** |  | -0.29 | 0.16 | 0.07 |  | -0.01 | 0.07 | 0.88 |
|  |  |  |  |  |  |  |  |  |  |  |  |  |  |  |
|  |  |  |  | TRIG | | |  | BUTY | | |  | NEFA | | |
| **juveniles** | df | Pillai’s Trace | multi- variate *p* | *β* | SE | uni- variate *p* |  | *β* | SE | uni- variate *p* |  | *β* | SE | uni- variate *p* |
| intercept |  |  |  | -0.27 | 0.11 |  |  | 0.04 | 0.14 |  |  | 0.57 | 0.09 |  |
| day of year | 1 | 0.10 | **< 0.001** | 0.01 | 0.003 | **< 0.001** |  | -0.01 | 0.003 | **< 0.001** |  | -0.004 | 0.002 | **< 0.01** |
| year 2017 | 1 | 0.09 | **< 0.001** | -0.18 | 0.06 | **< 0.01** |  | -0.12 | 0.07 | 0.15 |  | -0.03 | 0.05 | 0.77 |
| body mass | 1 | 0.22 | **< 0.001** | 0.02 | 0.004 | **< 0.001** |  | -0.01 | 0.01 | 0.08 |  | -0.01 | 0.003 | **< 0.001** |
| bleed time | 1 | 0.07 | **< 0.01** | 0.00 | 0.001 | **< 0.01** |  | -0.01 | 0.002 | **< 0.001** |  | 0.002 | 0.001 | **< 0.01** |
| sin (time of day) | 1 | 0.04 | **0.03** | -0.21 | 0.07 | **< 0.001** |  | 0.11 | 0.10 | 0.18 |  | 0.07 | 0.06 | 0.17 |
| cos (time of day) | 1 | 0.03 | 0.098 | 0.11 | 0.05 | **0.02** |  | -0.07 | 0.06 | 0.25 |  | -0.03 | 0.04 | 0.47 |
| species: | 3 | 0.37 | **< 0.001** |  |  | **< 0.001** |  |  |  | **< 0.001** |  |  |  | **0.02** |
| pectoral sandpiper |  |  |  | 0.17 | 0.10 |  |  | -0.69 | 0.13 |  |  | -0.16 | 0.08 |  |
| semipalmated plover |  |  |  | 0.14 | 0.09 |  |  | -0.58 | 0.12 |  |  | -0.18 | 0.07 |  |
| semipalmated sandpiper |  |  |  | 0.43 | 0.08 |  |  | -0.85 | 0.10 |  |  | -0.05 | 0.06 |  |

| **Supplementary Table 8.** Parameter estimates and standard errors from final models examining the effect of drought (measured by the Standardized Precipitation and Evapotranspiration Index, SPEI) on the mass of shorebirds at James Bay, Ontario, Canada. Blank cells indicate terms that were not included in initial models or were removed from final models because they were not significant predictors. Estimates are presented in the log scale and listed in reference to adults at the mean day of year (if applicable) and mean SPEI. Significant effects (*α* = 0.05) are bolded; *p* was < 0.04 for all significant terms. | | | | | |  |
| --- | --- | --- | --- | --- | --- | --- |
|  | predictor *β* ± SE | | | | |  |
| species | intercept (adult) | day of year | SPEI | juvenile | juvenile by SPEI |  |
|  |  |  |  |  |  | *R^2^* |
| least sandpiper | 3.14 ± 0.01 | **0.001 ± 0.0003** | 0.04 ± 0.01 | **-0.08 ± 0.01** | **-0.04 ± 0.01** | 0.04 |
|  |  |  |  |  |  |  |
| semipalmated sandpiper | 3.33 ± 0.001 | **0.01 ± 0.0001** | **0.02 ± 0.001** | **-0.18 ± 0.001** | **-0.005 ± 0.002** | 0.38 |
|  |  |  |  |  |  |  |
| white-rumped sandpiper | 3.81 ± 0.003 | **0.01 ± 0.0003** | **0.04 ± 0.004** |  |  | 0.32 |
|  |  |  |  |  |  |  |
| semipalmated plover | 3.79 ± 0.005 |  | **0.03 ± 0.005** | **-0.07 ± 0.007** |  | 0.15 |
|  |  |  |  |  |  |  |
| pectoral sandpiper females | 4.27 ± 0.01 |  | **0.03 ± 0.01** |  |  | 0.01 |
| males | 4.61 ± 0.01 | **0.004 ± 0.001** | **0.02 ± 0.01** |  |  | 0.06 |

**Supplementary Figures**


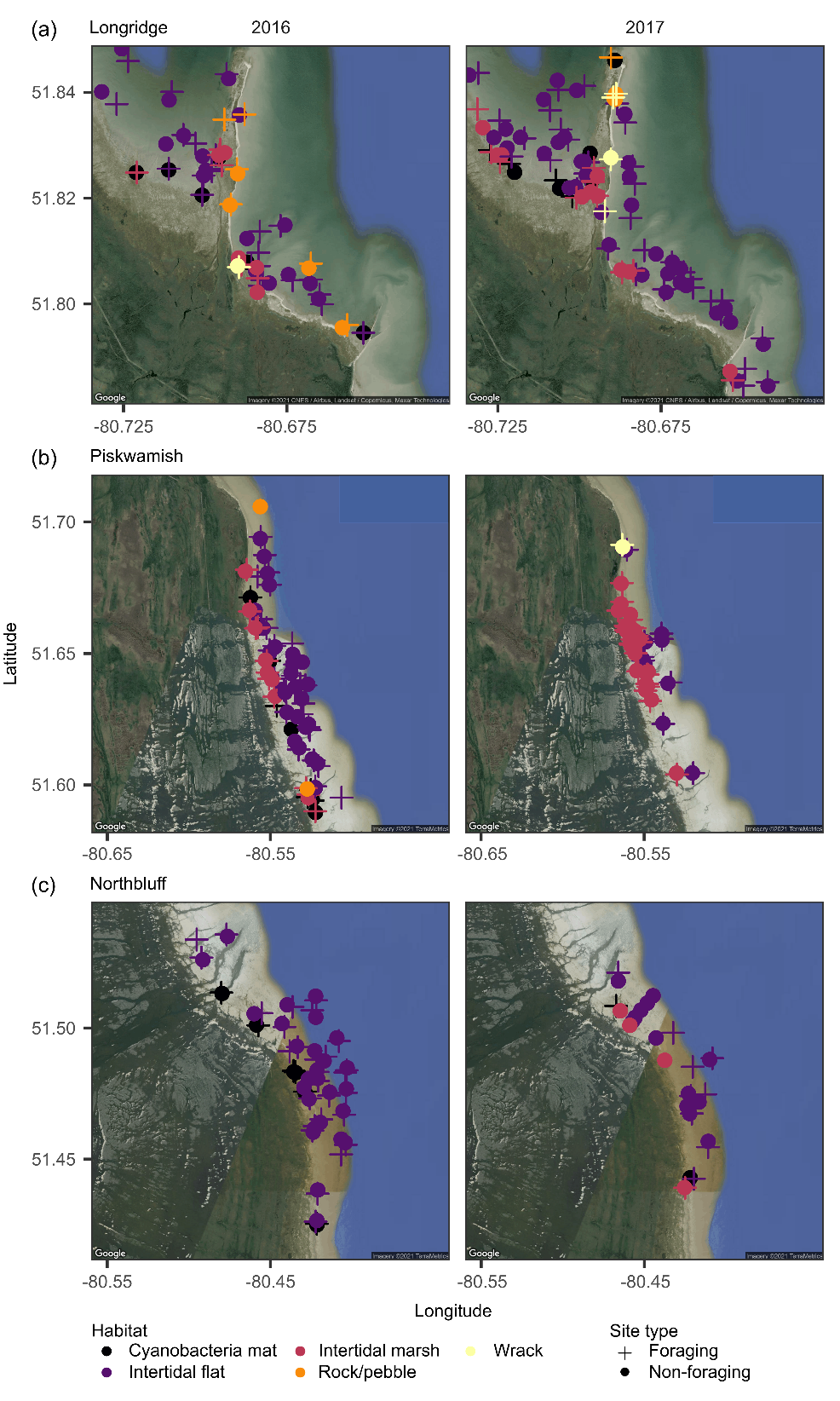


**Supplementary Figure 1.** Habitat types and locations of benthic invertebrate sample sites at three field camps (a) Longridge, (b) Piskwamish, and (c) Northbluff along James Bay, Ontario, Canada in 2016 and 2017. Each point designates a site where three benthic cores were collected at sites where birds were foraging or at non-foraging sites. We did not take enough samples from three habitats that covered small patches in the study area (wrack: *n* = 11; rock/pebble: *n* = 16; cyanobacteria mat: *n* = 43), so they were excluded from the formal analyses in the paper.


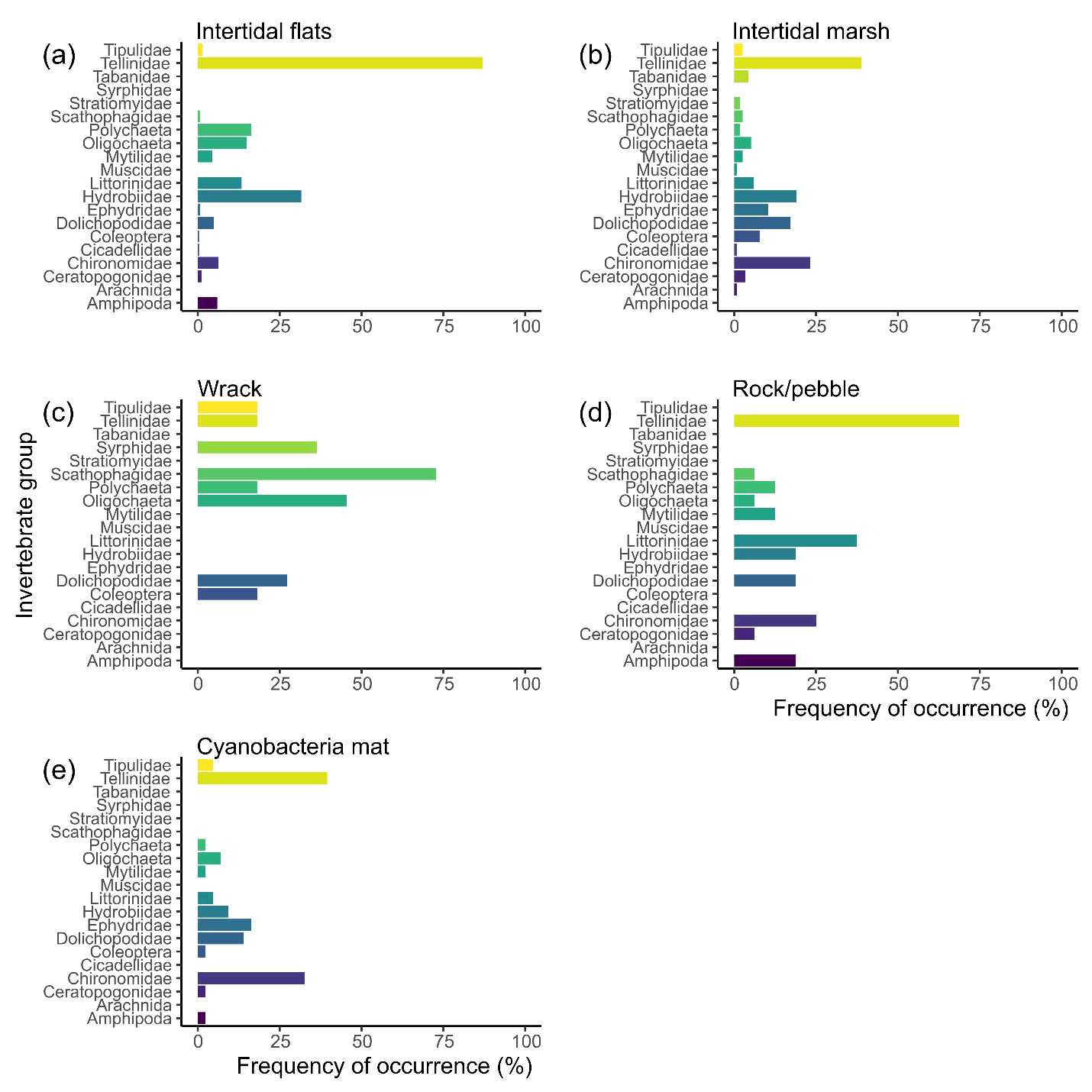


**Supplementary Figure 2.** Frequency of occurrence of invertebrate groups identified at benthic core sampling sites during 2016 and 2017 along the southwestern coast of James Bay, Ontario, Canada. Frequency of occurrence was calculated for different sample sizes in each habitat type (a) intertidal flats: *n* = 269; (b) intertidal marsh: *n* = 116; (c) wrack: *n* = 11; (d) rock/pebble: *n* = 16; (e) cyanobacteria mat: *n* = 43).


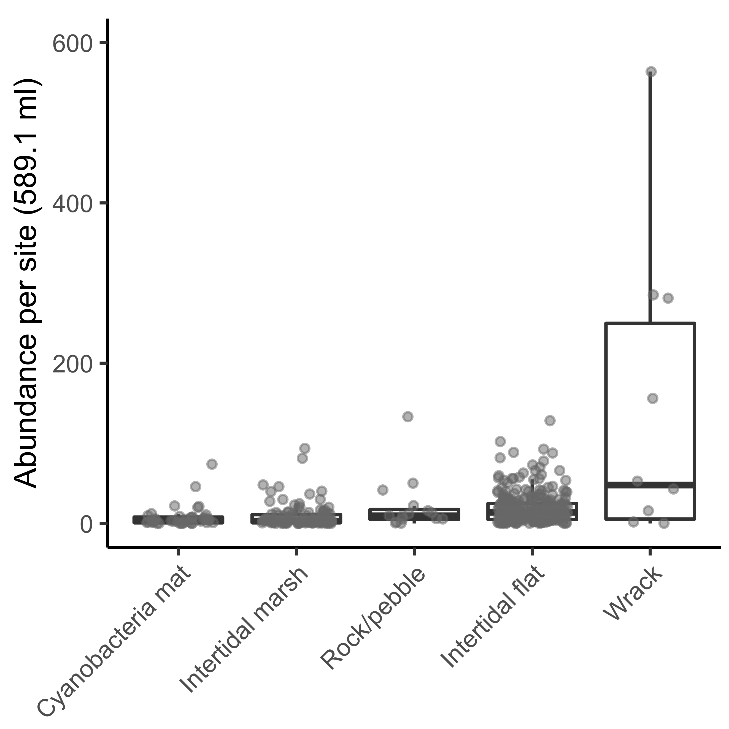


**Supplementary Figure 3.** Benthic invertebrate abundance data by habitat type for samples collected in 2016 and 2017 along the southwestern coast of James Bay, Ontario, Canada. For simple representation, each point is total invertebrate abundance from three replicate cores taken at each sampling site (bird or control). Boxplots are ordered by median invertebrate abundance.

One wrack site contained 3,925 invertebrates (almost entirely small oligochaetes) per 589.1 ml and was not plotted in this figure.


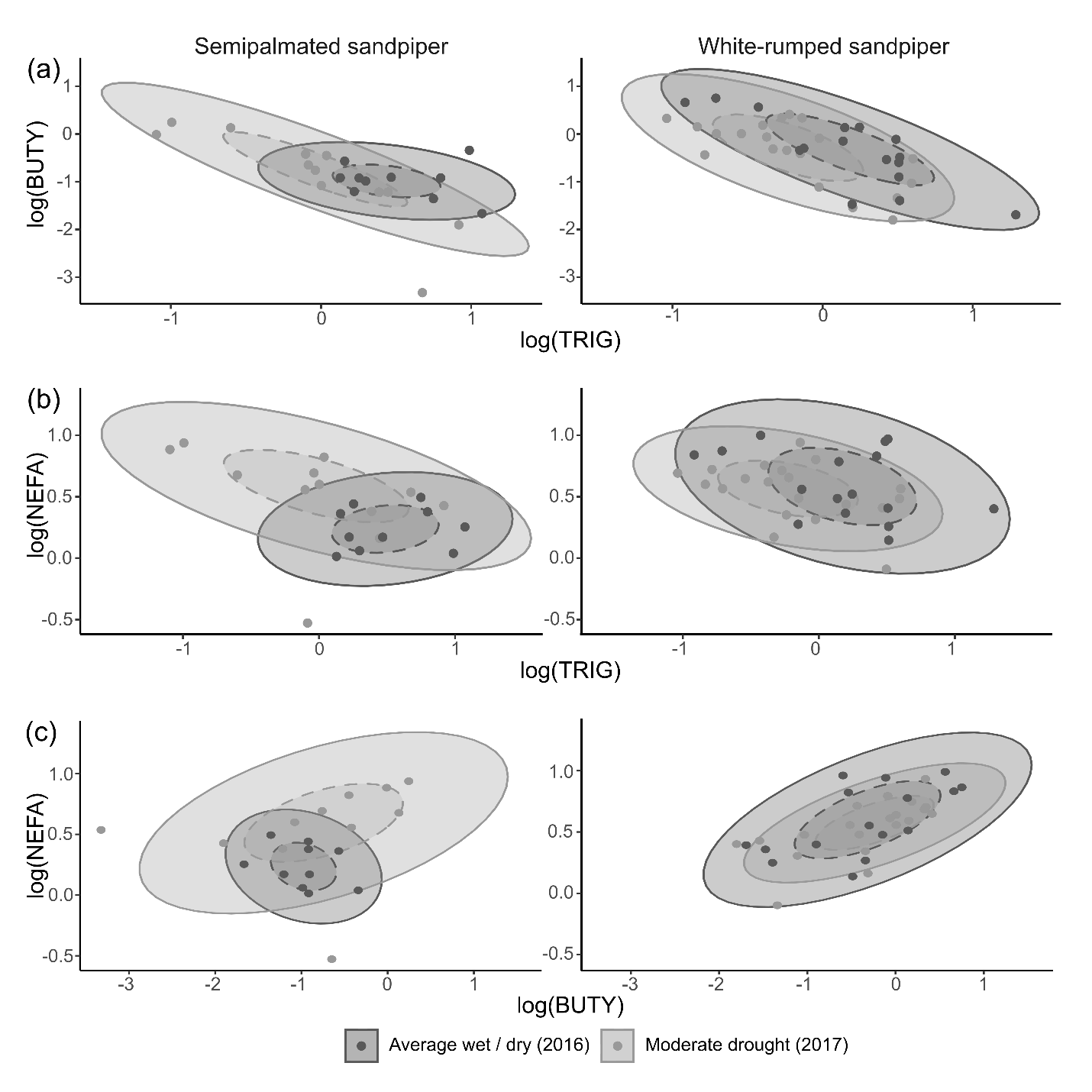
**Supplementary Figure 4.** Relationships between three plasma metabolites for adult shorebirds captured at James Bay, Ontario, Canada in 2016 and 2017. Solid lines designate 95% data ellipses, whereas dashed lines designate 50% data ellipses.


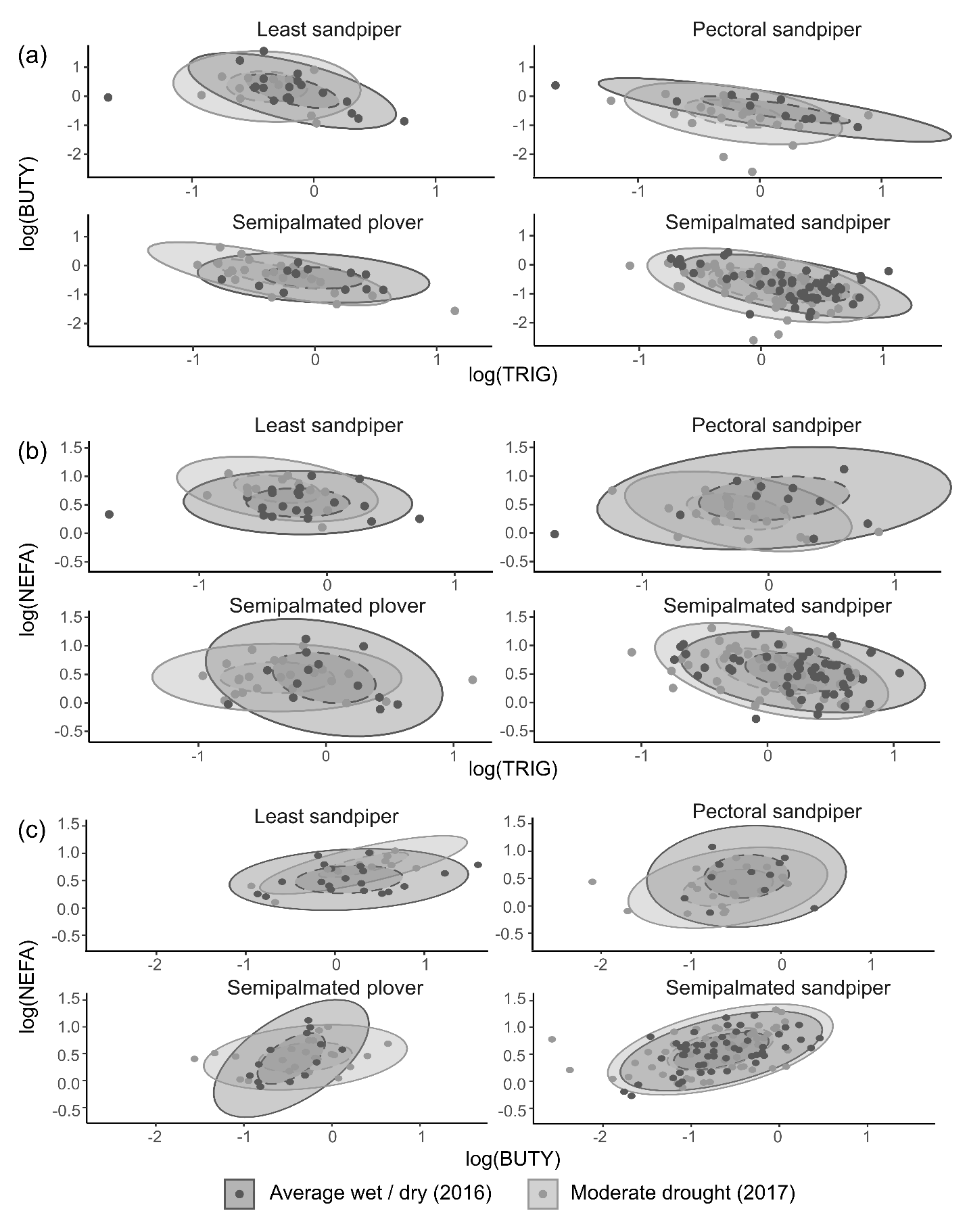


**Supplementary Figure 5.** Relationships between three plasma metabolites for juvenile shorebirds captured at James Bay, Ontario, Canada in 2016 and 2017. Solid lines designate 95% data ellipses, whereas dashed lines designate 50% data ellipses.


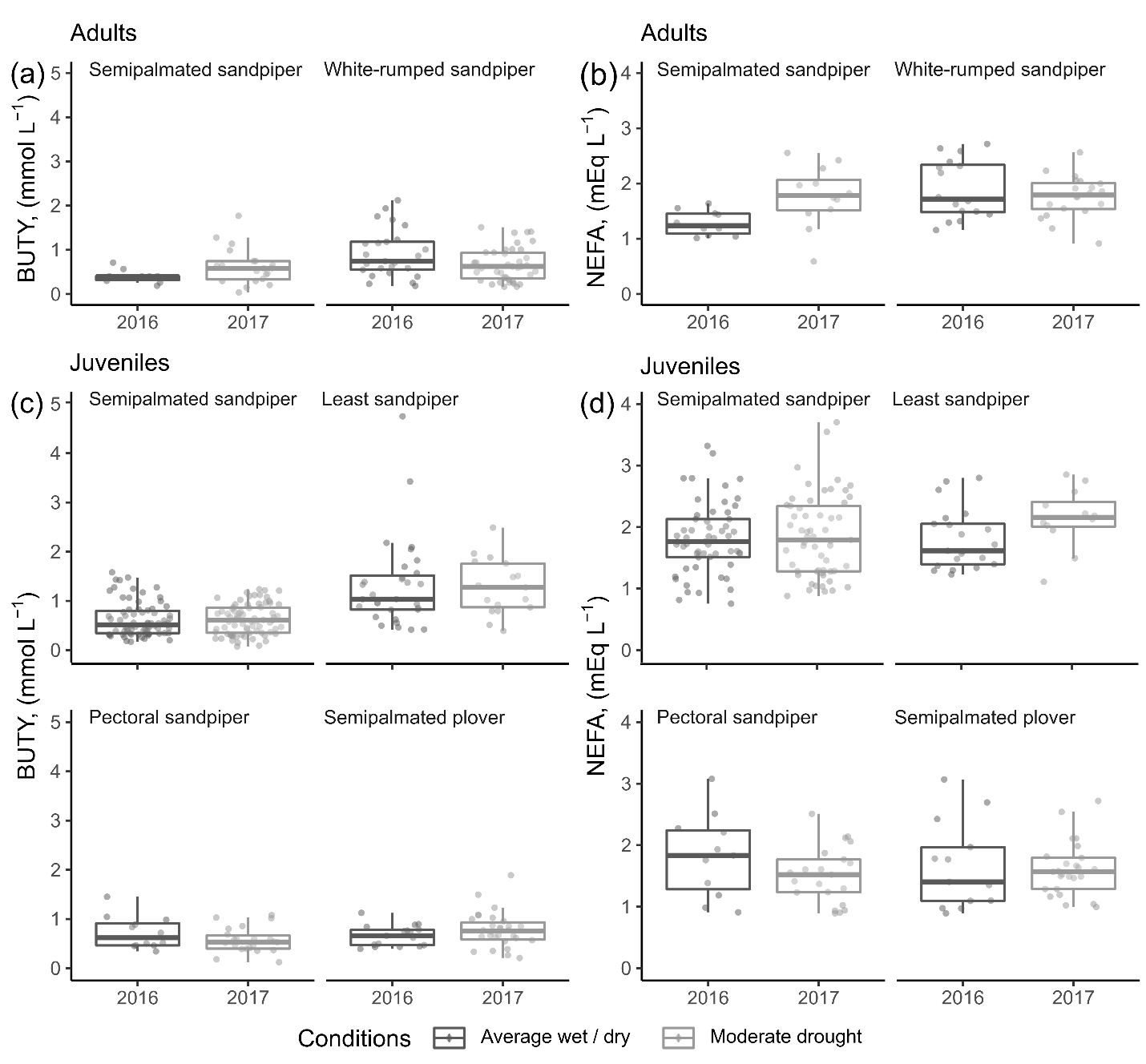


**Supplementary Figure 6.** Plasma BUTY and NEFA concentrations for shorebirds at stopover along the southwestern coast of James Bay in a year with average wet/dry conditions (2016) and a year with moderate drought (2017). Boxplots and points represent raw data. There was no significant difference between years in univariate tests (*α* = 0.05)


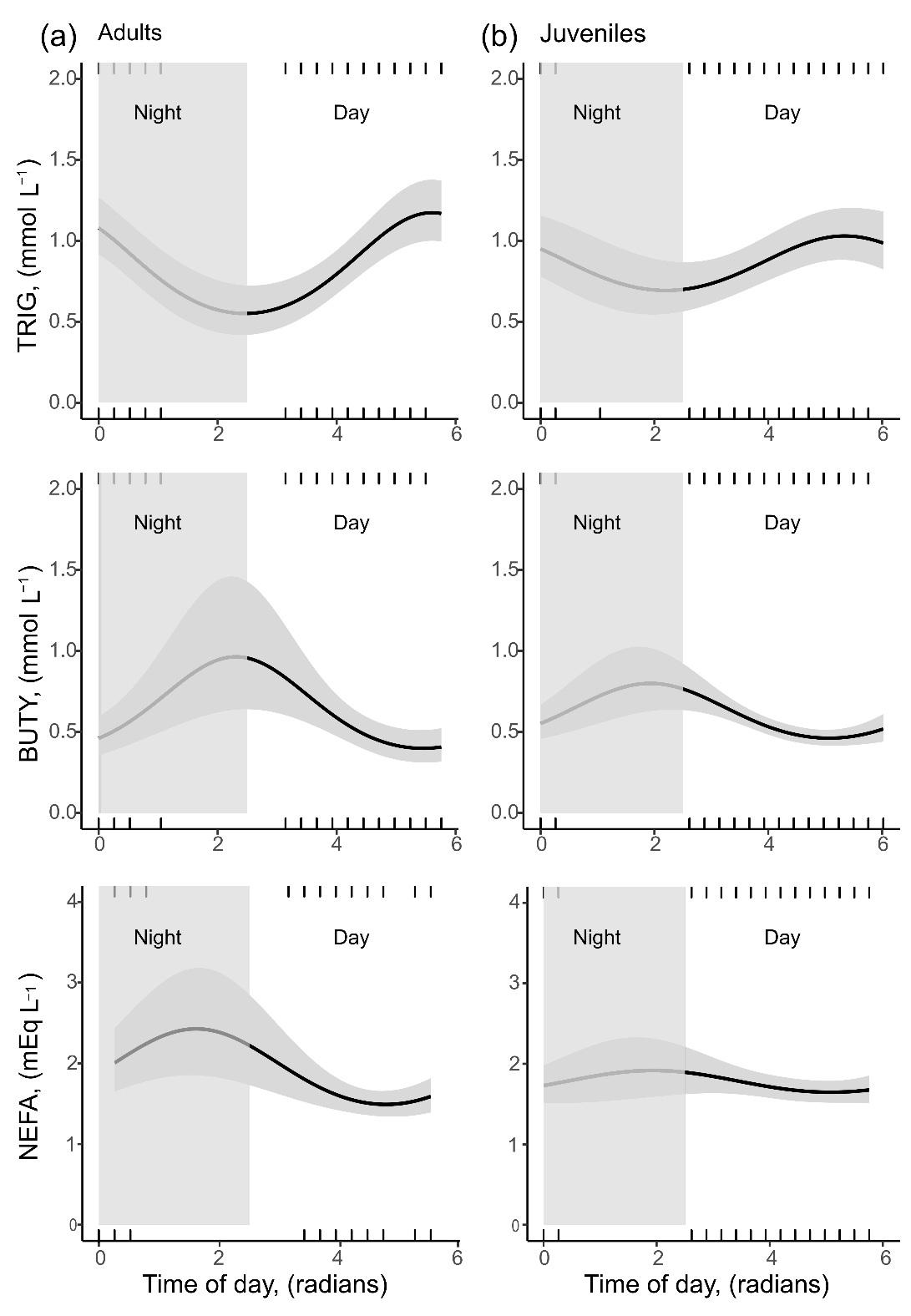


**Supplementary Figure 7.** Daily patterns in plasma metabolite concentrations for (a) adult and (b) juvenile shorebirds during stopover at James Bay, Ontario, Canada during 2016 - 2017. Trends plotted are from post-hoc univariate models. The grey panel corresponds to night: the time of civil twilight (0 radians, 21:00 EDT) to civil dawn (2.5 radians, 5:30 EDT) on 16-Aug, the mean day of year for all samples.


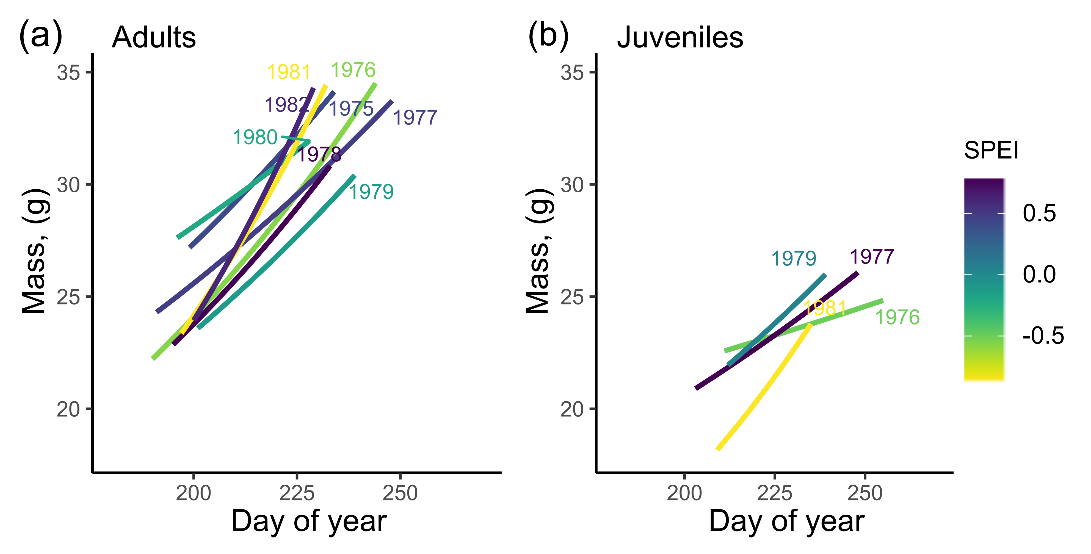


**Supplementary Figure 8.** Mass gain of (a) adult and (b) juvenile semipalmated sandpipers recaptured at James Bay, Ontario, Canada during southbound migration in the historical monitoring period (1975 – 1982; adults 1975: *n* = 77, 1976: *n* = 373, 1977: *n* = 447, 1978: *n* = 228, 1979: *n* = 91, 1980: *n* = 21, 1981: *n* = 30, 1982: *n* = 46; juveniles 1976: *n* = 148, 1977: *n* = 210, 1979: *n* = 68, 1981: *n* = 30). We only included individuals captured > 5 days apart in the analysis because individuals tended to lose weight for up to five days after capture (Gratto 1983). The slope of the lines represents the average rate of mass gain for semipalmated sandpipers for a given year. The rate of mass gain depended on the year-specific 3-month SPEI value for adults (χ^2^ = 6.4, d.f. = 1, *p* = 0.01) but not juveniles (χ^2^ = 1.9, d.f. = 1, *p* = 0.17), though we only had 4 years of recapture data for juveniles. For adults, mass at initial capture was lower, but rates of mass gain were higher in drier years, indicating that adults were able to reach high masses at James Bay even during years of moderate drought (1981). We compared rates of mass gain among years using a linear mixed effects models with mass as the response variable (log transformed) and 3-month SPEI, year, day of year, and a year-specific SPEI by day of year interaction. We included bird ID as a random factor and ran separate models for juveniles and adults.
